# Evolving role of CD8^+^ tissue-resident memory T cells in autoimmune and immune mediated inflammatory diseases: linking pathogenesis to therapeutic approaches

**DOI:** 10.3389/fimmu.2026.1903750

**Published:** 2026-09-07

**Authors:** Fatemeh Sadat Tabatabaei, Shannon K. Bromley

**Affiliations:** Center for Immunology and Inflammatory Diseases, Division of Rheumatology, Allergy and Immunology, Massachusetts General Hospital, Harvard Medical School, Boston, MA, United States

**Keywords:** adaptive immunity, autoimmunity, CD103, CD49a, CD69, chronic inflammation, skin disease, T cells

## Abstract

Memory CD8^+^ T cells provide accelerated protection against intracellular pathogens and tumors. Circulating memory T cells recirculate through blood and lymphoid tissues and can enter peripheral tissues during infection, whereas tissue-resident memory T cells (T_RM_), persist long-term in peripheral tissues, often at sites of previous pathogen infection where they can rapidly respond to antigen re-exposure. Although CD8^+^ T_RM_ cells lodge long-term within tissues, they are not stationary; many survey their local environment. CD8^+^ T_RM_ cells respond rapidly to antigen encounter by producing cytokines. These cytokines stimulate epithelial and innate immune cells to produce antimicrobial peptides and chemokines promoting recruitment of circulating leukocytes. Their localization within tissues provides protection against local reinfection, but persistent or dysregulated CD8^+^ T_RM_ cells may also sustain tissue inflammation and promote local disease recurrence in autoimmune and immune-mediated inflammatory diseases. Here, we review the mechanisms that establish and maintain CD8^+^ T_RM_ cells; evaluate evidence for their pathogenic roles in inflammatory disease; and discuss current and emerging therapeutic strategies for targeting pathogenic CD8^+^ T_RM_ cells.

## Introduction

### An overview of CD8^+^ T_RM_ cells

**Figure 1:**
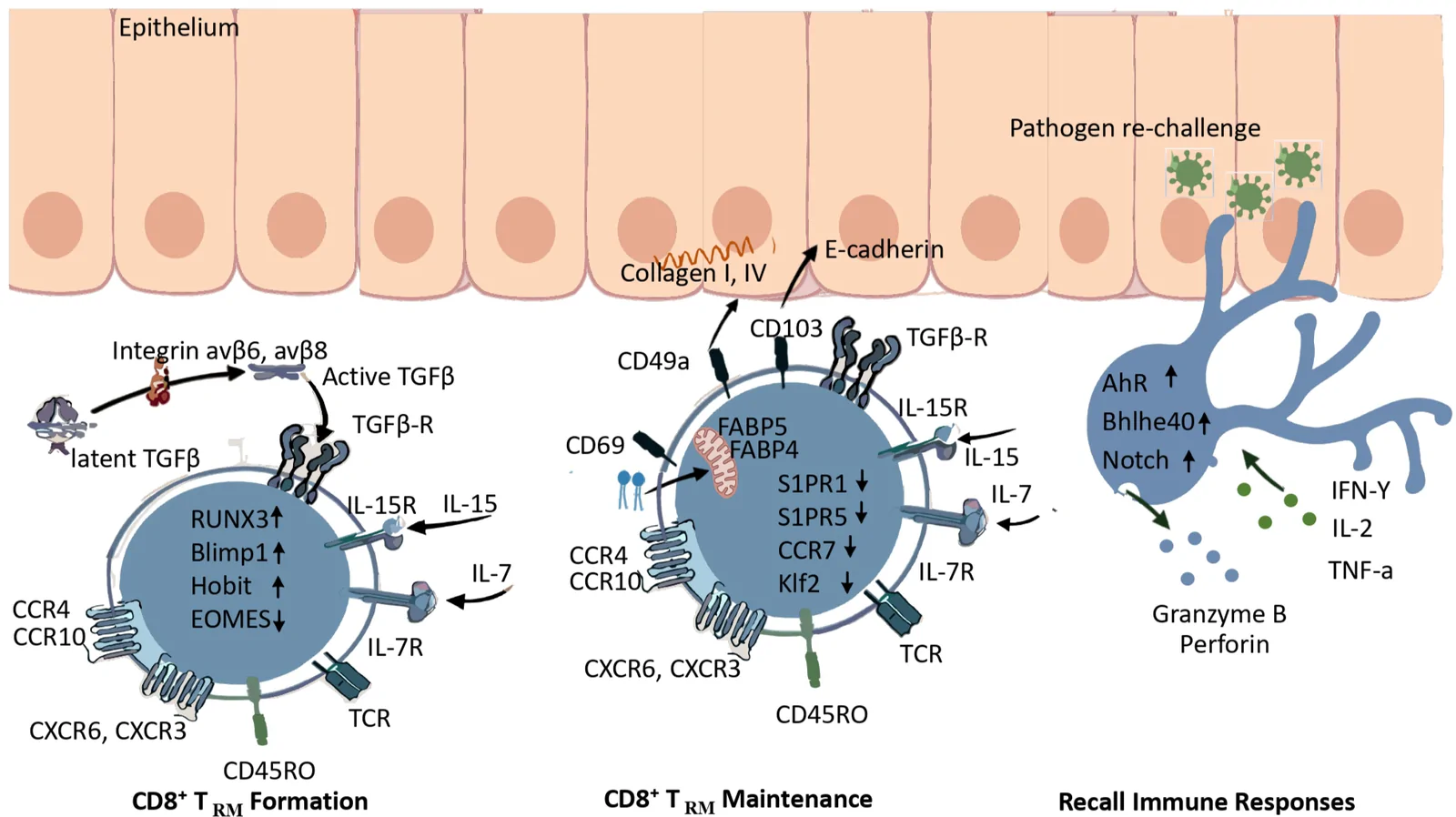
Overview of CD8^+^ tissue-resident memory T (TRM) cell formation, maintenance, and recall responses in the skin. Local cytokines including TGF-β promote the establishment of the TRM transcriptional program, while suppressing tissue egress pathways. Chemokine receptors facilitate tissue homing and positioning. TRM survival is further sustained by IL-7/IL-15 signaling and lipid metabolism pathways involving FABP4 and FABP5. Upon pathogen re-challenge, resident CD8 TRM cells rapidly produce effector molecules.

CD8^+^ tissue-resident memory T cells (T_RM_) can persist within peripheral tissues for many months, and in some settings, for more than one year, independently of circulating memory T cells (1–3). In several infection models, they provide more rapid local protection against infection compared with circulating memory CD8^+^ T cells. Upon antigen recognition, CD8^+^ T_RM_ cells rapidly produce interferon-γ (IFN-γ) and tumor necrosis factor-α (TNF-α), and can release cytotoxic molecules such as granzyme B. CD8^+^ T_RM_ cell-derived cytokines activate local innate immune cells and induce chemokine and adhesion molecule expression, which promotes recruitment of circulating immune cells (4–6). After antigen re-exposure, CD8^+^ T_RM_ cells can proliferate *in situ* (7) (**Figure 1**). While their residence within tissues permits a rapid response to local reinfection, persistent or dysregulated CD8^+^ T_RM_ cells can also contribute to chronic or relapsing autoimmune and inflammatory diseases.

### Surface markers used to identify CD8^+^ T_RM_ cells

CD8^+^ T_RM_ cells are transcriptionally and phenotypically distinct from circulating memory CD8^+^ T cells. CD103 and CD69 are the markers most frequently used to identify CD8^+^ T_RM_ cells. Although CD69 is transiently expressed by all CD8^+^ T cells after activation (8), CD8^+^ T_RM_ cells often maintain its expression (9–12). CD69 binds S1PR1 and promotes its internalization (13). Reduced S1PR1 expression limits responsiveness to S1P gradients that promote lymphatic egress from peripheral tissues (14).

Expression of CD69 and CD103 varies both between tissues and among CD8^+^ T_RM_ cells within the same tissue. While epithelial CD8^+^ T_RM_ cells express CD103 (15–17), CD8^+^ T_RM_ cells in several tissues (small intestinal lamina propria, kidney, liver, and lymph nodes) may lack CD103 and rely on other adhesion molecules for retention or local migration (18–22). For example, the integrin, LFA-1 mediates the patrolling behavior by CD8^+^ T_RM_ cells within liver sinusoids (23), and CD103^-^CD69^+^CD8^+^ T cells recovered from human intestinal transplants highly express β2-integrin (24). Parabiosis studies have demonstrated that the functional requirement of CD69 expression for tissue residency also varies between tissues (25). Because CD69 is also induced transiently after T cell activation (8), it should not be used alone to define T_RM_ cells.

### Egress and recirculation of CD8^+^ T_RM_ cells

Fonseca et al. tested whether reactivated skin CD8^+^ T_RM_ cells can exit the tissue and reenter the circulation (26) They generated immune mice harboring cutaneous antigen-specific CD8^+^ T_RM_ cells, and then depleted circulating memory CD8^+^ T cells—but not cutaneous CD8^+^ T_RM_ cells. They then challenged the mice with cognate antigen. Antigen-specific CD103^+^CD49a^+^CD62L^-^ CD8^+^ T cells were subsequently identified within the blood, draining and distant lymph nodes, suggesting that they originated from the skin. After restimulation, their progeny also showed a preferential tendency to return to the tissue from which they originated (26). Together, these findings suggest that reactivated skin CD8^+^ T_RM_ cells can exit the tissue, circulate, and retain a bias for entering the skin.

## Transcriptional regulators control CD8^+^ T_RM_ cell formation, residency and response

### Repression of tissue egress

CD8^+^ T_RM_ cell persistence requires inhibition of tissue egress together with signals that support retention and survival. The transcription factor, KLF2 maintains expression of receptors important for T cell circulation including S1PR1, CCR7 and CD62L (27). After entry into peripheral tissues, CD8^+^ T_RM_ cells downregulate KLF2 and S1PR1, thereby limiting their responsiveness to S1P exit signals. Consistent with this role, forced expression of S1PR1 reduced CD8^+^ T_RM_ cell establishment in multiple tissues following LCMV infection (28). PI3K signaling decreased KLF2 expression by inhibiting FOXO1, a KLF2 transcriptional activator (29). Because several cytokines activate PI3K, local cytokine signals may regulate KLF2 expression through the PI3K-FOXO1 pathway. FTY720 (fingolimod), a functional S1PR1 antagonist, traps T cells in lymph nodes (30, 31), thereby limiting new seeding of tissues with CD8^+^ T_RM_ precursor cells. S1PR1^lo^ CD8^+^ T_RM_ cells may be relatively unaffected (32).

HOBIT and BLIMP-1 further suppress genes associated with tissue egress. HOBIT and BLIMP-1 cooperate to bind and repress *Klf2*, *Ccr7* and *S1pr1* (33, 34). Their requirements differ between tissues. *Zfp683 (Hobit)*-deficient mice exhibited impaired CD8^+^ T_RM_ cell formation in skin and liver (33). However, BLIMP-1, not HOBIT, was required for CD8^+^ T_RM_ cell differentiation in the lung (35). The importance of HOBIT in human CD8^+^ T_RM_ cells remains unclear, because it is also expressed by circulating effector and effector memory CD8^+^ T cells (36).

RUNX3 is required for CD8^+^ T_RM_ cell formation and maintenance in multiple tissues (37). *Runx3* deficiency reduced CD8^+^ T_RM_ cells, while RUNX3 overexpression in CD8^+^ T cells induced CD103 and CD69, suppressed *Klf2*, *Ccr7*, *S1pr1*, and increased chromatin accessibility at TGF-β-responsive genes, including *Itgae* (CD103) (37). Although few CD4^+^ T_RM_ cells are present in epithelial tissues at homeostasis, aberrant expression of RUNX3 in CD4^+^ T cells promoted CD4^+^ T_RM_ cell residency in skin and intestinal epithelium (38).

### Local cues link transcription factor expression and residency

Local cytokine cues regulate transcription factors that promote CD8^+^ T cell persistence and survival. TGF-β activates SMAD signaling and cooperates with RUNX3 to induce expression of genes such as *Itgae* (CD103), *Itga1* (CD49a), and *Cd69* (37–41). TGF-β also induces Notch ligand expression by epithelial cells (42). Notch receptor expression is induced by IL-2, inflammatory cytokines, and TCR signaling (43), and these receptors can be activated by epithelial Notch ligands. Peripheral blood CD8^+^ T cells cultured *in vitro* with TGF-β and Notch ligand expressed increased CD103 compared to cells cultured with either factor alone, suggesting that TGF-β and Notch might cooperatively regulate CD103 expression (44). *In vivo*, γ-secretase inhibition, which inhibits Notch signaling, decreased numbers of established lung CD8^+^ T_RM_ cells and downregulated their expression of integrins and nutrient/ion transporters, suggesting Notch regulates CD8^+^ T_RM_ cell persistence and metabolism (45).

BHLHE40 and AhR integrate inflammatory and environmental signals that support CD8^+^ T_RM_ cell differentiation and persistence. BHLHE40 is induced by inflammatory cytokines, IL-15, PGE2 and costimulatory signals, and it regulates CD8^+^ T_RM_ cell formation, maintenance and polyfunctionality. *Bhlhe40*^-/-^ mice had fewer CD103^+^ CD8^+^ T_RM_ cells in the lung following influenza infection and exhibited decreased effector molecule expression compared to WT mice. RNA-seq and functional analyses showed that BHLHE40 regulates genes involved in mitochondrial metabolism and oxidative phosphorylation (46). Programmed cell death protein 1 (PD-1) signaling suppressed BHLHE40 expression in tumor-infiltrating lymphocytes (TILs), and BHLHE40 was crucial for anti-PD-L1 therapy efficacy (47).

The aryl hydrocarbon receptor (AhR) is a ligand-activated transcription factor that responds to ligands produced by the microbiome, diet, and cellular metabolic processes (48). AhR promoted CD8^+^ T_RM_ cell differentiation, while suppressing acquisition of a T_CM_ phenotype (49). Although *Ahr*^-/-^ CD8^+^ T cells initially entered inflamed skin, they were unable to persist within the epidermal niche (50). Similarly, *AhR*^-/-^ CD103^+^CD69^+^CD8^+^ T cells failed to persist in the intestine and expressed decreased Granzyme B compared to WT CD8^+^ T_RM_ cells (49).

These findings indicate that CD8^+^ T cell residency depends on coordinate transcriptional regulation of tissue retention, survival and metabolic adaptation.

## Tissue-derived signals: cytokines, inflammatory molecules, and other immune cell signals

### Chemokines

Chemokines direct CD8^+^ T cells into tissues and their subsequent localization within microenvironments that influence CD8^+^ T_RM_ cell differentiation, maintenance and function. Tissue-selective migration depends on the chemokine receptors expressed by circulating T cells and the ligands displayed within target tissues. For example, intestine-homing CD8^+^ T cells express CCR9, and respond to CCL25, constitutively expressed by small intestinal endothelial cells (51, 52). In contrast, skin-homing CD8^+^ T cells express CCR4 (53) and CCR10 (54) whose ligands CCL17 and CCL27, respectively, are displayed by dermal microvascular endothelium. These chemokine pathways promote tissue-specific surveillance at distinct barrier sites.

Inflammation induces expression of additional chemokines that recruit CD8^+^ T_RM_ precursor cells. For example, following vaginal herpes simplex virus infection, CD4^+^ T cell-derived IFN-γ induces CXCL9 and CXCL10 expression and recruitment of CXCR3^+^ CD8^+^ effector T cells into the infected tissue (55) and subsequent local CD8^+^ T_RM_ cell establishment (56). CXCL9 and CXCL10 are also often expressed in chronically inflamed and autoimmune tissues and may contribute to the recruitment of circulating cells into tissues containing pathogenic CD8^+^ T_RM_ cells.

Chemokines also guide CD8^+^ T cell positioning within tissues. In the lung, CXCR3 directs CD8^+^ T cells into the CXCL9- and CXCL10-rich airway regions (57), whereas CXCR6-CXCL16 signaling promotes the establishment and maintenance of airway CD8^+^ T_RM_ cells (58). In the skin, CXCL16 produced by dermal myeloid cells supports CXCR6^+^ CD8^+^ T_RM_ precursor cell survival (59, 60) before their CXCR3-dependent entry into the epidermis (16). The requirement for these pathways varies by tissue compartment and inflammatory context. For example, while CXCR3 is important for CD8^+^ T cell recruitment into the epidermis, it is not required for subsequent motility of epidermal CD8^+^ T_RM_ cells (61).

Chemokine-dependent positioning within tissue niches influences the cellular interactions and local signals that support CD8^+^ T_RM_ cell differentiation, persistence and function. Spatial analysis of the intestine showed that CXCR3 directs CD8^+^ T cells within niches containing different concentrations of TGF-β, IL-7 and IL-15, thereby influencing their acquisition of resident-memory characteristics (62).

### Cytokines

Local cytokines regulate CD8^+^ T_RM_ cell differentiation, survival, and effector function in distinct tissue microenvironments. TGF-β is required for the differentiation and prolonged maintenance of CD8^+^ T_RM_ cells in epithelial tissues, where it induces expression of retention receptors such as CD103 and CD49a (39). In the epidermis, TGF-β activation by integrins αvβ6 and αvβ8 is essential for CD8^+^ T_RM_ cell differentiation and persistence (63). CD8^+^ T_RM_ cells themselves produce TGF-β, and competition for active TGF-β may contribute to the selective enrichment of antigen-specific CD8^+^ T_RM_ cells within the epidermis (64).

IL-15 promotes CD8^+^ T_RM_ cell survival, proliferation and expression of cytotoxic effector molecules, including expression of perforin and granzyme B (65). However, cytokine requirements vary by tissue. In the small intestine and reproductive tract, CD8^+^ T_RM_ cells appear to rely less heavily on IL-15 (66). CD8^+^ T_RM_ cell survival and adaptation are regulated by the interplay between IL-15 and IL-7 signaling pathways. Crosstalk between IL-15 signaling and IL-7R expression may allow CD8^+^ T cells to adapt to changes in local IL-7 and IL-15 availability (67).

Local signals also contribute to CD8^+^ T_RM_ cell functional heterogeneity (68). In human epidermis, CD49a^+^CD103^+^ CD8^+^ T_RM_ cells were enriched for cytotoxic functions and production of IFN-γ, perforin, and granzyme B. Conversely, CD49a^−^CD103^+^ CD8^+^ T_RM_ cells preferentially produced IL-17 (65). However, CD49a does not always predict effector function across tissues or inflammatory diseases (69). Therefore, surface phenotype should be interpreted together with transcriptional and functional evidence.

### Other immune cell signals: CD4^+^ T-cell help

CD4^+^ T cells influence memory CD8^+^ T cell priming, tissue recruitment and CD8^+^ T_RM_ cell differentiation. During priming in the lymph node, CD4^+^ T cells license dendritic cells via CD40-CD40L and promote cytokine production that supports CD8^+^ T cell survival and recall responses (70, 71). In peripheral tissues, CD4^+^ T cells can also promote CD8^+^ T_RM_ cell establishment by producing IFN-γ and inducing chemokines that recruit effector CD8^+^ T cells into sites of infection (55) where they can differentiate into T_RM_.

CD4^+^ T cells provide local help for CD8^+^ T_RM_ cell differentiation (72). During polyomavirus infection, CD4^+^ T cell depletion did not impair the initial accumulation, proliferation, or cytokine production of brain-infiltrating CD8^+^ T cells, but reduced their differentiation into CD103^+^ T_RM_ cells. In CD4-deficient mice, few brain CD8^+^ T cells upregulated CD103, and these “unhelped” cells showed elevated PD-1, became dependent on continuous resupply from the circulation, and displayed downregulated Runx3. Ren et al. (73) showed that this CD4-dependent effect was mediated in part by IL-21 signaling through IL-21R. IL21R-deficient brain CD8^+^ T cells failed to upregulate CD103 and mounted impaired recall responses. These cells remained dependent on replenishment from the circulation and showed reduced expression of oxidative-metabolism genes which was restored by IL-21 supplementation. Local CD4^+^ T cell help and IL-21 signaling also determined the magnitude of the antigen-specific CD8^+^ T_RM_ cell response in the lung (74).

CD4^+^ T cell help may also regulate the differentiation of established or precursor CD8^+^ T_RM_ cells into pathogenic effector cells. In a CNS autoimmunity model, CD4-depletion did not eliminate the TCF-1^+^ CD8^+^ T_RM_ precursor pool, but did prevent its differentiation into TCF-1^-^ cytotoxic effectors (75, 76).

## Metabolic programming of CD8^+^ T_RM_ cells: from lipid uptake to function

Long-term survival of CD8^+^ T_RM_ cells depends in part on their ability to use nutrients available within the local tissue environment. In the skin, CD8^+^ T_RM_ cells support their survival through uptake and oxidation of exogenous free fatty acids. The fatty acid binding proteins (FABPs) FABP4 and FABP5 are essential for maintaining cutaneous CD8^+^ T_RM_ cell populations following VACV infection and contribute to their protective function during viral challenge (77). However, FABP usage differs across tissues. Following LCMV infection, liver CD8^+^ T_RM_ cells predominantly express *Fabp1*, with some *Fabp4* and minimal *Fabp5* expression. Similarly, siIEL CD8^+^ T_RM_ cells express *Fabp1*, *Fabp2*, and *Fabp6*, while showing little to no expression of *Fabp4* and *Fabp5*. FABP expression therefore varies by tissue and may reflect local metabolic demands (78).

## CD8^+^ T_RM_ cells in autoimmune and immune-mediated inflammatory diseases

Although CD8^+^ T_RM_ cells provide rapid local protection, they may also contribute to autoimmune and inflammatory disease. CD8^+^ T_RM_ cells may sustain local inflammation and promote recurrence through their cytokine production, cytotoxic activity and interactions with tissue-resident and recruited immune cells (5). Chemokines that recruit CD8^+^ T_RM_ precursor cells are also expressed in chronically inflamed tissues and may support the establishment and maintenance of pathogenic tissue-resident immune responses (79, 80).

In the following sections, we review evidence supporting the role of CD8^+^ T_RM_ cells in autoimmune diseases and selected immune-mediated inflammatory disorders. Autoimmune diseases are characterized by adaptive immune responses directed against self-antigens, whereas immune-mediated inflammatory diseases involve pathogenic immune activation without a confirmed self-antigen target.

### Skin

#### Vitiligo

Vitiligo is an autoimmune disease in which autoreactive CD8^+^ T cells accumulate in the skin and target melanocytes, the cells responsible for pigment production (81–85). Upon discontinuation of therapy, depigmented lesions often re-develop at the same anatomical sites, suggesting the presence of persistent local immune memory (86). Melanocyte-specific CD8^+^ T cells recognizing MelanA/Mart1, tyrosinase and gp100 have been detected in vitiligo, and their frequency correlated with disease activity (87, 88). However, in a mouse model of tumor-associated vitiligo, melanocyte-specific CD8^+^ T_RM_ cells populated the skin broadly but were preferentially enriched in hair follicles containing depigmentation, sites where the target antigen had already been eliminated by prior melanocyte destruction (89). Thus, melanocyte-specific CD8^+^ T cells may persist in skin even after local antigen loss, suggesting that antigen dependence may vary among vitiligo-associated CD8^+^ T cell populations.

Lesional CD8^+^ T cells express markers associated with a T_RM_ cell phenotype, including CD49a, CD103, and CD69 (65, 90, 91). Transcriptomic analyses showed that non-lesional vitiligo skin had an intermediate expression profile between lesional vitiligo skin and healthy control skin. This intermediate profile was particularly apparent for inflammatory genes, especially those associated with IFN-γ responses (92). However, whether the CD8^+^ T cells in normal-appearing skin are resident remains unknown; CD8^+^ T cell clusters with similar transcriptional identities were present within both never-lesional and peri-lesional vitiligo skin, arguing against the idea that non-lesional skin is simply free of autoreactive resident cells. Pathways associated with immune activation were enriched in peri-lesional CD8^+^ T cells, whereas never-lesional CD8^+^ T cells showed enrichment for immune-regulatory pathways, lower type 1 cytokine expression, and higher PD-1 expression, accompanied by greater regulatory T cell infiltration in never-lesional skin (93).

Studies in mice indicate that skin-resident CD8^+^ T_RM_ cells are not sufficient on their own to maintain established vitiligo. In a mouse model, treatment with FTY720 to block entry of recirculating memory T cell into the skin, or selective depletion of this population, reversed disease despite persistence of CD8^+^ T_RM_ cells in the skin. These findings indicate that resident and recirculating memory T cells cooperate to maintain established vitiligo (94).

CXCR3 signaling contributes to recruitment of circulating CD8^+^ T cells into vitiligo skin. CD8^+^ T cells residing in vitiligo lesional skin highly expressed CXCR3 (94–97), and CXCL9 and CXCL10 expression was significantly increased in these regions compared to both non-lesional skin from affected patients and skin from healthy individuals (84, 95). Circulating CXCR3^+^ CD8^+^ T cells were also significantly increased in patients with vitiligo, both in those with active disease and in those in remission, compared to individuals without vitiligo (96). Circulating levels of CXCL9 and CXCL10 were elevated in patients with active vitiligo compared to those in remission and to healthy individuals (84, 96). During treatment, decreases in circulating CXCL10 correlated with clinical improvement, whereas changes in CXCL9 did not show a consistent association (95).

CXCL9 and CXCL10 were also increased in murine models of vitiligo (98). Vitiligo did not develop in CXCR3-deficient mice, suggesting that CXCR3 may be essential for autoreactive T cell accumulation in the skin to initiate the autoimmune response (95). An IFN-γ-driven circuit may sustain autoreactive CD8^+^ T cell accumulation in vitiligo skin. IFN-γ secreted by lesional CD8^+^ T cells drove keratinocyte production of CXCL9 and CXCL10, which may promote further recruitment of CXCR3^+^ CD8^+^ T cells into the skin. Mouse studies showed distinct roles for the two chemokines: CXCL9 promoted initial recruitment of autoreactive T cells to the skin, whereas CXCL10 was required for their localization and effector function after entry. Disruption of either CXCR3 or CXCL10 was sufficient to prevent depigmentation, supporting a functional role for this pathway in vitiligo (95).

IL-15 has also been implicated in the maintenance of melanocyte-specific CD8^+^ T_RM_ cells. Compared to non-lesional skin, lesional keratinocytes in patients with vitiligo expressed increased IL-15Rα (CD215), which enables trans-presentation of IL-15. Melanocyte-reactive CD8^+^ T_RM_ cells expressed higher levels of CD122, the β chain of the IL-15 receptor complex, than non–melanocyte-specific CD8^+^ T cells (91). Thus, maintenance of established vitiligo appears to involve both skin-resident and recirculating CD8^+^ memory T cells, with CXCR3 signaling contributing to recruitment and IL-15 promoting local CD8^+^ T_RM_ cell persistence.

#### Alopecia areata

Alopecia areata (AA) is an autoimmune skin disease that targets hair follicles (99). AA is characterized by collapse of hair-follicle immune privilege and aberrant MHC class II and adhesion molecule expression. Th1-derived IFN-γ promotes the recruitment of cytotoxic CD8^+^ T cells to the perifollicular region, resulting in hair cycle arrest (100). IL-15 signaling through the JAK1/JAK3 pathway also promotes IFN-γ production in CD8^+^ T cells, while IFN-γ contributes to collapse of hair follicle immune privilege. The effects of IL-15 in AA may differ between immune and epithelial compartments. While the number of perifollicular IL-15-producing T cells was increased in AA lesional skin, expression of IL-15 and its receptor within the hair bulb epithelium itself was down-regulated. Restoring local IL-15Rα-dependent signaling with recombinant IL-15 prevented IFN-γ-induced collapse of hair follicle immune privilege and promoted hair regrowth in an experimental human skin model (101). Thus, the effects of IL-15 in AA appear to be context dependent, with potentially distinct functions in follicular epithelial cells and infiltrating lymphocytes.

Although the autoantigens driving the CD8^+^ T-cell response in AA remain incompletely defined, a study using synthetic epitopes derived from the candidate hair follicle autoantigens trichohyalin and tyrosinase-related protein-2 (TRP2) demonstrated that cytotoxic T lymphocytes from patients with AA exhibited significantly greater IFN-γ responses than those from healthy controls (102). Reactivity to trichohyalin-derived epitopes was proposed as a potential prognostic marker (103).

Evidence that lesional T cells can mediate AA comes from adoptive-transfer studies in which T cells isolated from patient AA lesions were transferred into immunodeficient mice. These mice subsequently developed features of hair loss. Histological examination of the affected areas revealed findings closely resembling AA in the original patients, including patchy destruction of hair follicles (104). Lesional CD8^+^ T cells exhibited increased cytotoxic effector activity (105). ITGAE expression was increased in lesional AA skin (100), and compared with non-lesional skin, lesional skin contained a higher frequency of CD8^+^ T cells expressing CD103 and CD69 (106). Lesional skin also showed increased IFN-γ signaling, JAK–STAT pathway activation, and expression of CXCL9 and CXCL10 (106).

Single-cell RNA sequencing of murine and human AA skin resolved CD8+ T cells along an “effectorness gradient”, progressing from naive and early activated states to cytotoxic and TRM populations. In the mouse model, selective depletion of CD8+ T cells, but not CD4+ T cells, NK cells, B cells, or γδ T cells, was sufficient to prevent and reverse disease in this model (107). However, this depletion does not distinguish the contribution of the resident skin population from that of circulating CD8+ T cells. In patients with AA, clonally expanded CD8^+^ T cells expressing CD103 and CD69 persisted within lesional sites after therapy was discontinued. These cells may contribute to AA recurrence (108), potentially explaining why lesions often reappear after discontinuation of treatment (109).

#### Systemic sclerosis

Systemic sclerosis (SSc), also referred to as scleroderma, is an autoimmune condition marked by inflammation, vascular abnormalities, and progressive fibrosis (110). CD8^+^ T cells recognizing a 15-amino-acid peptide spanning residues 505–519 of DNA topoisomerase I (Scl-70), a major autoantigen in SSc, have been identified in patients with SSc (111).

A predominant population of CD8^+^ T cells in SSc skin mostly lacked CD103, but strongly expressed CD69 and CCR10. A subset of these cells were capable of producing granzyme B, IFN-γ, and IL-13, a cytokine with profibrotic functions that may contribute to disease pathogenesis (110). Granzyme B and perforin were also detected in SSc lesions (112–114), suggesting that these CD8^+^ T cells may contribute to local tissue injury in addition to producing IL-13.

A separate study found increased frequencies of CD8^+^CCR10^+^, CD8^+^CLA^+^, and particularly CD8^+^CCR4^+^CCR10^+^ T cells in the blood of patients with SSc compared with healthy controls. In addition, these CD8^+^ T cells expressed IL-13, granzyme B, and perforin (115). However, these features alone do not establish a tissue-resident phenotype, as circulating effector and effector-memory T cells can also express skin-homing receptors and produce effector cytokines. Furthermore, although immunohistochemistry demonstrated abundant CD8^+^ IL-13^+^ cells within dermal infiltrates during the early inflammatory phase of disease, late-stage lesions contained few IL-13^+^ cells and were predominantly populated by CD4^+^ T cells (115). Single-cell transcriptomic and epigenomic profiling of SSc skin identified an early effector-memory CD8^+^KLRB1^+^IL7R^+^ subset with cytolytic and Tc2/Tc17 effector programs, which predominated in early-stage diffuse cutaneous SSc lesions and directly induced tissue damage and fibrosis in ex vivo skin explants. Later stage lesions were instead enriched for an exhausted-like CD8^+^KLRG1^+^IL7R^−^ subset. RNA velocity and pseudotime trajectory analysis linked these populations along a common trajectory, consistent with a transition from an early cytolytic/profibrotic phenotype toward a later exhausted phenotype (116).

In the lungs of patients with SSc-associated interstitial lung disease (SSc-ILD), CD8^+^ T_RM_-like cells were identified, characterized by increased expression of CD69, ZNF683 (HOBIT), and ITGAE, along with low expression of S1PR1. Compared to healthy lungs, the proportion of CD8^+^ T_RM_-like cells was expanded (117). Trajectory analysis suggested that resting CD8^+^ T cells progressively differentiate into CD8^+^ effector memory and then CD8^+^ T_RM_-like populations in SSc-ILD lungs, a transition the authors propose was driven by a global T cell stimulus (117).

#### Cutaneous lupus erythematosus

Systemic lupus erythematosus (SLE) is a chronic autoimmune disorder characterized by multi-organ involvement, including the kidneys and the central nervous system (118). Although the precise antigen specificities of skin-infiltrating T cells in CLE remain largely undefined, studies in SLE have identified T cells reactive to nucleosomes and histones (119), and these responses have been linked to anti-dsDNA antibody production (120).

CLE lesions exhibit a prominent IFN-response signature, with activation of JAK/STAT signaling and increased expression of genes induced by type I and type II IFNs. IFN-induced chemokines, such as CXCL9, CXCL10, and CXCL11, promoted recruitment of CXCR3-expressing T cells, particularly CD8^+^ T lymphocytes, into lesional skin. Infiltrating T cells produced granzyme B and IFN-γ, and IFN-γ could further induce CXCL9, CXCL10 and CXCL11 expression in skin (121).

T cells expressing CD69 or CD103 were more abundant in CLE lesional skin than in control skin. The increase was independent of concomitant systemic disease, and CD8^+^ T_RM_-like cell abundance did not correlate with lesional severity in this cohort (122). In contrast, another study reported a positive correlation between CD8^+^CD103^+^ T cell abundance and disease severity (123). Thus, the relationship between local CD8^+^ T_RM_-like cell burden and clinical severity remains uncertain. Of note, patients with CLE who exhibited resistance to antimalarial therapies, one of the standard treatments for this condition (124), were found to harbor increased numbers of CD69^+^CCR7^+^ T cells compared with treatment responders. However, the study did not determine whether these cells were predominantly CD4^+^ or CD8^+^ T cells. The investigators proposed that CCR7 expression in these cells may be relatively reduced compared with circulating T cells rather than completely absent. Thus, CD69 expression alone is insufficient to establish that this population is tissue resident, particularly given the retained CCR7 expression (125).

IL-17 has been reported to be increased in CLE lesional skin, with CD8^+^ T cells identified as one potential source. However, the study did not establish that the IL-17-producing CD8^+^ T cells were tissue resident (126). In contrast, transcriptomic profiling of human lesional skin did not identify an elevated IL-17 signature relative to healthy skin (127).

#### Psoriasis

Psoriasis is a chronic skin disease characterized by erythematous scaly plaques (128). Although it is conventionally classified as an immune-mediated inflammatory disease rather than a classical autoimmune disease, several defined autoantigens have been identified, and antigen-specific, clonally expanded T cell populations have been described. In addition, increasing evidence implicates CD8^+^ T_RM_ cells in disease recurrence.

Several candidate autoantigens have been described including: a disintegrin and metalloproteinase with thrombospondin motifs-like protein 5 (ADAMTSL5), produced by dermal melanocytes, presented via the psoriasis susceptibility allele HLA-C*06:02 and recognized by IL-17–producing CD8^+^ T cells (129); the antimicrobial peptide LL37, which is overexpressed in psoriatic skin and recognized by both CD4^+^ and CD8^+^ T cells (130); and neolipid antigens generated by mast-cell-derived phospholipase (131). Consistent with antigen-driven selection, deep sequencing of the T cell receptor repertoire in clinically resolved psoriatic lesions identified oligoclonal populations of IL-17–producing αβ T cells bearing shared, psoriasis-specific TCR sequences that were absent from healthy skin. The same clones were detectable across multiple resolved lesions from the same patient. These results are consistent with MHC-restricted antigen-driven expansion of disease-associated T cell clones.

Psoriatic lesions improve with treatment; however, upon discontinuation of therapy, they reappear at the same sites and approximately at their pre-treatment size. Studies of clinically resolved psoriatic lesions have shown only a modest reduction of dermal CD8^+^ T cells after treatment. The phenotype of these residual CD8^+^ cells was not fully resolved, and it remains unclear whether they are effector memory or T_RM_ cell subsets (132).

In active psoriatic lesions, the hyperplastic epidermis contained up to 100-fold more CD8^+^ T_RM_ cells than healthy skin, with IL-17–producing T_RM_ cells particularly expanded (65). Notably, even in clinically resolved psoriatic skin, where total CD8^+^ T_RM_ numbers largely normalized to levels comparable to healthy skin, a specific subset of CD8^+^CD103^+^CCR6^+^IL-23R^+^ T_RM_ cells poised for IL-17 production remained selectively enriched. Thus, a disease-associated CD8^+^ T_RM_ subset persisted despite clinical resolution (133).

Further functional studies have demonstrated that engraftment of non-lesional skin samples from patients with psoriasis into immunodeficient mouse models induced psoriasis in those mice (134). Moreover, depletion of CD8^+^ T cells in this unaffected skin prior to xenotransplantation prevented lesion development (135). A randomized trial of an anti-E-selectin antibody, designed to block the recruitment of circulating T cells into skin via the E-selectin/cutaneous lymphocyte antigen interaction, found no efficacy in treating psoriasis (136). The lack of efficacy of E-selectin blockade is compatible with, but does not establish, a major contribution of tissue-resident T cells in sustaining psoriasis. In addition, activation of T cells in regions of skin that had never previously developed lesions induced psoriasis-specific transcriptional programs (137). These areas of skin are enriched in CD8^+^CD103^+^CD49a^−^ T cells with enhanced IL-17 production compared to healthy controls (137, 138). These studies provide evidence for antigen-experienced CD8^+^ T_RM_ cells in psoriatic skin and suggest that local memory populations may contribute to disease recurrence.

The following sections examine evidence for CD8^+^ T_RM_ cells in autoimmune diseases affecting organs beyond the skin and immune-mediated inflammatory diseases, where the literature remains comparatively limited.

### Intestine

#### Inflammatory bowel disease

Inflammatory bowel disease (IBD), including Crohn’s disease (CD) and ulcerative colitis (UC), generally follows a relapsing–remitting course (139, 140). Repeated local exposure of the intestinal mucosa to diverse luminal antigens may promote recurrent inflammatory flares (141). T_RM_ cells are abundant within the intestinal mucosa. Within the epithelium, they are predominantly CD8^+^ T cells, whereas both CD4^+^ and CD8^+^ T cell populations are present in the lamina propria (142). In colonic samples from patients with IBD, these cells express CD44, CXCR6, Hobit and Blimp-1, with reduced expression of S1PR1 and KLF2, consistent with a T_RM_ phenotype (143). However, the abundance and function of these cells vary by differentiation state, tissue compartment, and disease.

In CD, CD103 and KLRG1 define largely mutually exclusive, functionally distinct CD8^+^ T_RM_ subsets. CD103^+^CD8^+^ T cells, although present at similar frequencies to those in healthy controls, were more responsive to TCR restimulation. They underwent extensive transcriptional reprogramming and upregulated the Type 17-associated mediators IL-22, IL-26, and CCL20. KLRG1^+^ cells were selectively expanded in inflamed CD mucosa and exhibited greater cytotoxic and proliferative potential (144). Transcriptomic profiling further demonstrated that intraepithelial and lamina propria CD8^+^ T_RM_ cells are distinct. Lamina propria T_RM_ cells expressed a resident-memory program centered on survival and cytokine signaling, whereas intraepithelial T_RM_ cells were enriched for TCR signaling, NK-cell-associated cytotoxicity genes, and immunoregulatory interaction pathways with non-lymphoid (epithelial) cells. Within the lamina propria, the CD103^−^ CD8^+^ T_RM_ cells compartment segregated further into a distinct ITGB2 (integrin β2)-high subset co-expressing GZMK, KLRG1, and PD-1, phenotypically and transcriptionally separable from CD103^+^ T_RM_ cells, representing a candidate pathogenic subset. Inflammation also induced distinct transcriptional changes in intraepithelial and lamina propria CD8^+^ T_RM_ cells (145).

Not all studies demonstrate T_RM_ cell expansion in IBD. Reduced numbers of intraepithelial CD8^+^ T_RM_ cells have been reported in both CD and UC colonic tissues compared with healthy controls. Despite this numerical decline, the remaining CD8^+^ T_RM_ cells retained a similar CD69^+^CD103^+^ phenotype. Ex vivo analyses further demonstrated significantly reduced CD8^+^ memory T-cell responses against commensal bacteria in patients with CD and UC compared with healthy individuals. In an *in vitro* model of human T_RM_-like cells, these cells retained the ability to produce pro-inflammatory cytokines such as IFN-γ, TNF-α, and IL-17, but showed increased IL-10 production. These findings raise the possibility that some specific T_RM_ subsets may contribute to local immunoregulation and tissue homeostasis (146).

Longitudinal evidence has linked persistence of clonally expanded CD8^+^ T cells with postoperative disease recurrence. In a prospective surgical cohort of patients with CD, T-cell receptor repertoire sequencing of resected ileal mucosa identified a subgroup with high clonal expansion. All patients in this high-clonality subgroup developed endoscopic recurrence within six months. Clonality at the time of surgery predicted recurrence overall, and this high-clonality phenotype was attributable specifically to the CD8^+^ T cell compartment. These same TCR clonotypes were detected in the neo-terminal ileum six months later, and repertoire stability itself predicted recurrence. These findings demonstrate persistence of disease-associated CD8^+^ T cell clones after resection, although they do not establish whether these cells persist as tissue resident cells or recirculate before reappearing at the site of recurrence (147).

### Hepatobiliary system

#### Autoimmune hepatitis

Autoimmune hepatitis (AIH) is a chronic immune-mediated liver disease characterized by hepatocyte-directed inflammatory injury, interface hepatitis, and progression to fibrosis or cirrhosis if untreated.

CYP2D6 is the best-defined autoantigen in Type 2 AIH and the target of anti-liver kidney microsomal type 1 (anti-LKM1) antibodies. Early studies identified CYP2D6-specific CD8^+^ T cells in both the peripheral blood and liver of patients with AIH using HLA-A2 tetramer staining. The magnitude of these antigen-specific CD8^+^ T-cell responses correlated with disease activity and declined following treatment (148, 149).

CD69^+^CD103^+^CD8^+^ T cells were significantly enriched in the livers of patients with AIH compared to controls, and their frequency correlated with disease severity. These cells expressed a pro-inflammatory and cytotoxic profile, producing mediators such as IFN-γ, TNF-α, and granzyme B, and their numbers decreased following immunosuppressive therapy (150). E-cadherin was widely expressed on hepatocytes in AIH liver and localized near CD8^+^ T cells, providing a potential ligand for CD103-mediated interactions. This differs from murine liver CD8^+^ T_RM_ cells, in which LFA-1 rather than CD103 has been shown to mediate retention of T_RM_ cells within hepatic sinusoids (23). Whether CD103 has a functional role in retaining human hepatic CD8^+^ T_RM_ -like cells, particularly in inflammatory liver disease, remains to be determined (151). IL-15 and TGF-β, both capable of promoting CD8^+^ T_RM_ cell differentiation *in vitro*, were also increased in AIH liver (150). Additional cytokines present in the hepatic microenvironment may support memory T cell persistence. IL-2 has been implicated in secondary expansion of human hepatic memory CD8^+^ T cells (152), while hepatocyte-derived IL-7 can support survival of hepatic memory T cells (153). Whether either pathway contributes specifically to maintenance of CD8^+^ T_RM_ -like cells in AIH has not been established.

Transcriptomic analysis of AIH identified PRDM1 (encoding BLIMP1) as the only transcription factor differentially expressed across both circulating immune cell clusters and liver tissue, with upregulation specifically in CD8^+^ and NK cell populations and linked to granzyme B production and CCL5/CCR5-dependent chemotaxis. Cell-cell communication analysis identified CCL5 signaling through CCR1, CCR3 and CCR5 as a prominent interaction between CD8^+^ T cells and NK cells. CD8^+^ T cells were the principal CCL5 source. Because CCL5-CCR5 signaling promotes T cell survival, proliferation, cytotoxic function, and immune cell recruitment, increased CCL5 signaling could further amplify cytotoxic CD8^+^ T cell responses in AIH (154). However, this analysis was performed on peripheral blood CD8^+^ T cells cross-validated against liver bulk transcriptomes rather than on sorted liver-resident CD8^+^ T cells directly.

Together, these findings demonstrate prominent cytotoxic CD8^+^ T cell responses and features of hepatic tissue residency in AIH, as well as a defined CYP2D6-specific CD8^+^ T cell response in type 2 AIH. However, whether the autoreactive, antigen-specific CD8^+^ T cells overlap with the hepatic CD8^+^ T-cell population displaying a T_RM_-like phenotype has not yet been investigated.

#### Autoimmune cholangitis

Autoimmune cholangitis is an inflammatory disease characterized by bile duct inflammation and injury, as well as immune-mediated destruction occurring within the portal areas, leading to liver fibrosis. A murine model of autoimmune cholangitis demonstrated that a population of CD8^+^CD69^+^CD62L^−^ cells accumulated in the liver of these mice during the disease. These cells displayed high expression of residency and cytotoxicity-associated genes, including *Cxcr6*, *Prf1*, *Gzma*, and *Runx3*, and were not detected in the circulation. They upregulated Hobit while downregulating S1pr1. Their residency was functionally confirmed by parabiosis. Importantly, they localized in close spatial proximity to bile ducts *in situ* and their presence was associated with portal inflammation, bile duct damage, and fibrosis (155).

PDC-E2 provides a defined target for autoreactive CD8^+^ T cells in human disease. Using HLA-A2 tetramers, PDC-E2-specific CD8^+^ T cells were found to be enriched roughly tenfold in the liver relative to blood in patients with autoimmune cholangitis, and these cells were directly cytotoxic against antigen-pulsed target cells (156). A subsequent study showed that CD103^+^ CD8^+^ T_RM_ cells were the dominant population of PDC-E2-specific CD8^+^ T cells in the livers of patients with primary biliary cholangitis (157). Their long-term survival depends on NUDT1, an enzyme that limits oxidative nucleotide damage by hydrolyzing oxidized purine nucleoside triphosphates. Blockade of NUDT1 eliminated this T_RM_ population and alleviated cholangitis (157). Thus, human studies demonstrate enrichment of PDC-E2-specific CD8^+^ T cells within liver, while murine studies provide functional evidence linking antigen-reactive CD8^+^ T_RM_ cells to disease. Whether the same mechanisms are required for maintenance of pathogenic CD8^+^ T_RM_ cells in human disease remains unknown.

#### Primary sclerosing cholangitis

Primary sclerosing cholangitis (PSC) is a chronic immune-mediated cholangiopathy characterized by chronic inflammation and progressive fibrosis of the intrahepatic and extrahepatic bile ducts, leading to strictures, cholestasis, and ultimately cirrhosis. Although PSC is not generally considered a classical autoimmune disease, it is included here because both experimental and human studies implicate antigen-experienced CD8^+^ T cells with tissue-homing, antigen-responsive features in biliary inflammation.

The gut–liver axis hypothesis has been proposed to explain why PSC is frequently associated with UC. Mechanistic support for this hypothesis comes from a transgenic mouse model in which the same antigen (ovalbumin) was expressed in both gut epithelium and cholangiocytes. CD8^+^ T cells primed against this antigen in the gut-associated lymphoid tissue acquired an effector phenotype, including IFN-γ production, cytolytic activity, and upregulation of the gut-homing integrin α4β7 alongside integrin β1 and CXCR3, and migrated to the liver roughly twice as efficiently as naive CD8^+^ T cells. When these gut-primed CD8^+^ T cells encountered their cognate antigen on cholangiocytes, their transfer resulted in a portal, CD3^+^ mononuclear infiltrate and elevated liver enzymes in an antigen-dependent manner; mice lacking the shared antigen in bile ducts were fully protected from cholangitis despite equivalent gut priming. Chronic intestinal inflammation was independently shown to upregulate hepatic ICAM-1, VCAM-1, and CCL25 and activate liver DCs, which increased retention and antigen-driven proliferation of gut-primed CD8^+^ T cells and potentiated cholangitis (158). This study demonstrates that gut-primed, antigen-specific CD8^+^ T cells can induce cholangitis when their cognate antigen is expressed by cholangiocytes, and that biliary expression of the shared antigen is required for disease in this model. Intestinal inflammation further enhances this response by increasing hepatic adhesion molecule and chemokine expression and activating liver DCs. Notably, the experiment tracks CD8^+^ T-cell effector function over approximately one week and does not directly test whether these cells subsequently persist long-term as a non-recirculating resident population.

Endoscopic sampling of human bile ducts demonstrated that PSC is characterized by marked infiltration of neutrophils and CD8^+^ T cells, with biliary CD8^+^ T cells predominantly exhibiting a CD69^+^CD103^+^CD49a^+^ phenotype. Similar to the mouse model, these biliary CD8^+^ T cells display a combined gut- and liver-homing profile, including expression of CXCR6, CCR6, CCR9, and α4β7 integrin, together with the inflammation-associated chemokine receptor CXCR3. Biliary neutrophil infiltration was associated with CXCL8, whereas CXCL16 was associated with T cell enrichment. Biliary CD8^+^ T cells also produced IL-17 and IL-22, suggesting that this T_RM_-like population may contribute directly to local inflammation within the bile ducts (159). This human population, however, has been characterized by marker expression at a single timepoint, and a contribution from recruited circulating antigen-experienced CD8^+^ T cells cannot be excluded.

### Pancreas

#### Diabetes

Type 1 diabetes (T1D) results from autoimmune destruction of pancreatic insulin-secreting β cells (160). In patients newly diagnosed with T1D, CD8^+^ T lymphocytes were a major fraction of the immune infiltrate within pancreatic islets (161), and islet antigen–specific CD8^+^ T cells could be detected in their peripheral circulation (162). However, the diabetogenic ability of certain Tc1 clones correlated most closely with CXCL10 expression than with cytotoxicity or homing properties (163). Experimental studies using the non-obese diabetic (NOD) mouse model have demonstrated that CD8^+^ T cells contribute to β-cell injury both during the initial phases of disease progression and in the terminal effector phase (164–166).

In human T1D, T cells that infiltrate the pancreas and target β cells may acquire a phenotype similar to CD8^+^ T_RM_ cells, allowing them to persist within the tissue. For example, in a murine pancreatic islet allograft rejection model, CD8^+^ T cells that migrated into the pancreas expressed CD103 over time. These cells persisted within the pancreas during ongoing inflammation (167). In addition, studies examining pancreatic islet lesions from patients with T1D have identified infiltrating CD8^+^ T cells expressing both CD69 and CD103, consistent with a CD8^+^ T_RM_-like phenotype (168). However, previous studies have also demonstrated that even in healthy pancreas, CD8^+^ T cells with similar T_RM_-like phenotypes were present (169), indicating that their presence alone is not sufficient to establish causality in disease pathogenesis. Nevertheless, in T1D lesions, these CD8^+^ T cells also produced high levels of IFN-γ, IL-22, and IL-15, as well as expressed elevated levels of CXCL9 (168).

Functional evidence for a pathogenic role of pancreatic CD8^+^ T_RM_ cells has emerged from the NOD mouse model. Pancreatic T_RM_ cells accumulated progressively in NOD mice, preceding diabetic onset, and expressed the highest levels of FABP4 among pancreatic immune subsets. FABP4-deficient T_RM_ cells showed increased apoptosis and reduced accumulation in the pancreas. FABP4 also promoted CXCL10 production by pancreatic CD8^+^ T_RM_ cells. CXCL10 produced by these cells increased the motility and recruitment of circulating CD8^+^ T cells into the pancreatic islets. This finding provides a potential cellular source for the CXCL10 signal previously associated with diabetogenicity (163). Treatment with an anti-CD69 neutralizing antibody before recruitment of circulating CD8^+^ T cells delayed diabetic onset and reduced T1D incidence in NOD mice. Together with the FABP4 experiments, these findings support a functional contribution of CD69^+^ pancreatic T_RM_ cells to disease development (170).

### Renal

#### Lupus nephritis

Lupus nephritis is the renal manifestation of SLE and frequently relapses despite standard therapies (171). Although autoantibodies and complement are central to renal injury, T cells are the predominant immune cell population within inflamed lupus nephritis biopsies (172). In a murine model in which membrane-bound ovalbumin is expressed as a renal autoantigen on podocytes, autoreactive T cells progressively accumulated within the kidney and acquired CD69 and CD103, while circulating CD8^+^ T cells were rapidly lost following antigen re-encounter. Local antigen exposure alone was sufficient to induce a CD69^+^CD103^+^ phenotype, suggesting that tissue antigen can promote acquisition of CD8^+^ T_RM_ cell-associated features within the kidney (173).

Human studies support persistence of CD8^+^ T cells within the kidney, but also reveal substantial heterogeneity, including populations with tissue-resident, cytotoxic, proliferative, and potentially recirculating phenotypes. Persistent T-cell clonotypes have been detected within renal inflammatory infiltrates across serial biopsy samples from patients with lupus nephritis, but shared clonotypes identified in both peripheral blood and renal tissue suggest that some infiltrating CD8^+^ T cells may retain the capacity to recirculate (174). Deng et al. identified transcriptionally distinct PD-1^+^CD8^+^ T-cell subsets, including cytotoxic, interferon-responsive, proliferating, and migratory populations. PD-1 expression identified a highly activated rather than terminally exhausted CD8^+^ T-cell population in both patients with lupus nephritis and NZB/W F1 mice. PD-1 signaling restrained IFN-γ production through a STAT1–T-bet regulatory axis, whereas PD-1 agonism with a PD-L1 Fc fusion protein significantly reduced renal injury, proteinuria, and autoantibody production in lupus-prone mice (175). These findings identify the PD-1–STAT1–T-bet axis as an important regulator of renal CD8^+^ T-cell function.

Renal CD69^+^CD103^+^CD8^+^ T cells accumulate within lupus nephritis kidney tissue (123). Systemic CD8^+^ T cell cytotoxicity also increased during active disease. Patients experiencing flares had a larger fraction of circulating perforin- and granzyme B–producing CD8^+^ T cells than patients with quiescent disease (176). A distinct subset of cytotoxic CD8^+^ T cells expressing CD107a, a marker of degranulation, was enriched in peritubular regions and correlated with proteinuria (177). Their abundance correlated with clinical and histopathological indicators of disease severity, including serum creatinine levels, podocyte injury, and glomerulosclerosis (178). Renal CD8^+^ T cells exhibited substantial proliferative capacity, which may contribute to their persistence within the kidney. JAK-STAT signaling contributed to both effector function and proliferation of renal CD8^+^ T_RM_-like cells (123).

Renal CD8^+^ T cells may also shape local humoral autoimmunity. Single-cell transcriptomic and cell-cell interaction analyses predicted extensive signaling between granzyme K-producing CD8^+^ T cells and atypical B cells within tertiary lymphoid structures. Through IFN-γ- and IL-21-mediated signaling, granzyme K-producing CD8^+^ T cells were predicted to promote the differentiation of atypical B cells into locally expanded extrafollicular antibody-secreting cells enriched for complement-fixing IgG1 and IgG3 isotypes, suggesting a potential contribution of renal CD8^+^ T-cells to local B cell differentiation and autoantibody production in lupus nephritis (179).

Comparison of lupus skin and kidney illustrates the tissue-specific adaptation of CD8^+^ T cells. Lupus skin was relatively enriched in CD8^+^ T_RM_-like cells, whereas the kidney was enriched in cytotoxic CD8^+^ T-cell and NK-cell clusters. Moreover, kidney-infiltrating T cells expressed significantly higher levels of *GZMB* and *PRF1* than skin T cells, consistent with a more pronounced cytotoxic program. Thus, CD8^+^ T cells in lupus kidney exhibit a more strongly cytotoxic transcriptional program than those in lupus skin (180).

### Brain

Local tissue signals support CD8^+^ T_RM_ cell formation and persistence within the brain. Regulatory T cell-derived TGF-β drove upregulation of CD103 on CD8^+^ T cells and was required for their long-term retention in the brain: depleting Tregs did not prevent the expansion of effector CD8^+^ T cells in the periphery, but selectively impaired their ability to remain in brain tissue (181). Separately, reactive glia can promote acquisition of a CD8^+^ T_RM_-associated phenotype by CD8^+^ T cells. Co-culture of activated CD8^+^ T cells with primary glial cells drove upregulation of CD103, CD69, and CD127. This effect was lost when using PD-1-deficient T cells or when glial PD-L1 was blocked, suggesting that PD-L1--PD-1 signaling contributes to glia-induced acquisition of T_RM_-associated markers (182).

CD8^+^ T cells in the white matter of the brain include CD103^+^CD69^+^ and CD103^-^CD69^+^ populations. These cells exhibited low expression of cytolytic enzymes, as well as reduced expression of T-bet and EOMES. In contrast, they showed high expression of inhibitory receptors, including PD-1 and CTLA-4 (183). These findings suggest that CD8^+^ T cells within the white matter of the brain may exist in a tightly regulated state with restrained effector function. Consistent with a protective function, in a lupus mouse model, ablation of brain CD8^+^ CD44^high^CD62L^low^CD69^+^ T cells exacerbated neuropsychiatric lupus (184).

#### Multiple sclerosis

In MS, a chronic autoimmune disease affecting the central nervous system, infiltrating lymphocytes contribute to demyelination, neuroinflammation, and neuronal injury. Autopsy studies of MS brain lesions suggested that CD8^+^CD44^+^ (both CD103^+^ and CD103^−^) T cells co-expressed CD69, CD49a, and PD-1, but lacked S1PR1 and CCR7 expression. These cells also expressed CXCR6 and Ki-67, lacked detectable granzyme B production, and were found in close association with microglia in demyelinating lesions. The same study identified TGF-β, highly expressed in astrocytes within active early-stage MS lesions, as a local cue driving CD103 induction (185). However, studies investigating the expression of CD8^+^ T_RM_-associated phenotypic markers in MS have yielded conflicting results. Some studies have reported that, although CD8^+^ T cells exhibit high expression of CD103, they lack CD69 expression (186). In contrast, other studies have shown that in late progressive MS, these cells express CD69 but lack CD103 (187).

Spatially resolved single-cell profiling of chronic active MS lesions identified CD8^+^ T_RM_ cells enriched in a lesion-rim niche adjacent to glycoprotein NMB^+^ microglia displaying an interferon-responsive, lipid-laden state associated with chronic MS inflammation. Receptor-ligand analysis predicted interactions involving CXCL16-CXCR6 and CCL5-CCR1 axes, together with integrin and MHC I-associated interactions. CD8^+^ T cells with elevated IFNG expression were associated with increased STAT1 activity in neighboring glycoprotein NMB^+^ microglia. Functionally, IFN-γ impaired cholesterol efflux and promoted lipid droplet accumulation in microglia exposed to myelin debris. In an MS mouse model, genetically blocking microglial cholesterol export increased CD8^+^ and CD4^+^ T cell infiltration and worsened disease, while pharmacologically restoring efflux reduced both. The authors propose that inflamed, lipid-laden microglia may participate in a self-amplifying feedback loop that sustains T cell recruitment and retention (188).

Although CD20 is classically regarded as a B-cell marker and the therapeutic efficacy of B-cell depletion has highlighted its importance in MS, CD20 expression has also been identified in small subsets of T cells. A population of CD20dimCD8^+^CD69^+^ T cells has been identified in MS white matter lesions that expressed CXCR6 and granzyme B (189). A subsequent study examined myelin-reactive CD8^+^ T cells in MS. Myelin-specific CD8^+^ T cells were detected at similar frequencies in MS patients and HLA-matched healthy controls, but a significantly greater proportion of these cells displayed a memory phenotype and expressed CD20 in MS patients, suggesting prior antigen encounter (190).

More direct evidence that reactivated CD8^+^ T_RM_ cells can cause CNS pathology comes from a mouse model in which mice were engineered to re-express a neo-self-antigen in glial cells. Antigen reactivation triggered pre-established CD8^+^ T_RM_ cell expansion and progressive, compartmentalized neuronal damage (76). CD4^+^ T cell help was not required for the T_RM_ cells’ initial establishment, but was necessary for their expansion and full effector differentiation. The authors proposed that neuronal injury caused by reactivated CD8^+^ T_RM_ cells releases neuronal antigen that drains to secondary lymphoid tissue, where it primes naive CD4^+^ T cells that subsequently differentiate into local, tissue-resident helper cells within the CNS. These resident CD4^+^ helper cells then sustain CD8^+^ T_RM_ persistence and effector function. A TCF-1^+^ CD8^+^ T cell population with a predominantly T_RM_-like phenotype was also identified in human demyelinating lesions (75, 76).

Local tissue-derived cytokines may further regulate disease pathogenesis. In a murine model of chronic CNS inflammation, selective deletion of IL-33 in oligodendrocytes resulted in a marked reduction of CD8^+^ T-cell infiltration and persistence within the brain, thereby alleviating the overall disease severity (191). In the absence of IL-33, self-reactive CD8^+^ T cells displayed limited differentiation into TCF-1^low^ effector phenotypes, accompanied by a significant decrease in CD103^+^CD69^+^ T_RM_-like populations in the CNS (192, 193). In parallel, microglia shifted toward a homeostatic phenotype, with a notable reduction in disease-associated microglial (DAM) markers (191). Thus, oligodendrocyte-derived IL-33 was associated with both accumulation and effector differentiation of CNS CD8^+^ T cells and acquisition of a disease-associated phenotype by microglia.

### Joints

#### Rheumatoid arthritis

Rheumatoid arthritis (RA) is a chronic autoimmune disease with periods of flare and remission that leads to progressive damage of the articular surface. The tendency of flares to recur in a limited set of previously affected joints led to the hypothesis that T_RM_ cells may contribute to local disease recurrence. In mouse models of RA, CD8^+^ CD44^hi^CD62L^lo^CD69^+^CD103^+/-^ ST2^+^ T cells exhibited signs of long-term residency. These cells failed to migrate toward the egress chemokine CCL21 *in vitro* and their persistence within joints was unaffected by FTY720-mediated blockade of S1P-dependent lymphocyte egress. Additionally, they preferentially took up free fatty acids. *In vivo*, these cells persisted for over eight months during remission in joints previously involved in the active state of the disease, were unable to migrate between joints, and could trigger disease flare upon antigen re-exposure. Site-specific depletion of these T cells during remission attenuated subsequent arthritis flare as measured by histology (194).

CD69^+^CD103^±^ CD8^+^ T cells have been recovered from the synovial fluid of adult RA patients with a T_RM_-like phenotype, including increased CXCR6, CD49a, and PD-1, decreased S1PR1 and KLF2, and markedly elevated granzyme B and perforin compared with CD69^-^CD8^+^ T cells (195). Dysregulated cellular and humoral immunity in RA promotes the generation of pathogenic autoantibodies. Synovial CD69^+^CD8^+^ T cells have also been implicated in neutrophil activation and NET formation through a perforin-dependent mechanism. Because NETs provide a source of citrullinated antigens, this pathway could potentially contribute to local anti-citrullinated protein antibody (ACPA) responses (195). However, this study was conducted on synovial fluid. It remains to be determined whether human CD8^+^ T_RM_ cells persist in the joint tissue itself.

CyTOF and scRNA-seq resolved synovial CD8^+^CD69^+^CD103^+^ T cells into three transcriptionally distinct subsets. A cytotoxic T_RM_-like population was characterized by a gene program enriched for *GZMA*, *GZMB*, *GZMH*, *GZMK*, *NKG7*, *PRF1*, *ITGAE*, *PDCD1*, *LAG3*, and *CTLA4*, together with reduced expression of *SELL*, *KLF2*, and *KLF3*. A Treg-like population expressed *FOXP3*, *IL2RA* (CD25), and *IKZF2* (Helios), with low *IL7R* expression, yielding a transcriptional profile resembling regulatory T cells. Finally, a type 17-like population was defined by the transcription factors *RORA*, *AHR*, *BATF*, and *ZNF683* (Hobit), accompanied by increased expression of *KLRB1*, *CCR6*, *CXCR6*, *IL17A*, *IL21*, and *IL26*, and decreased expression of *S1PR1* and *EOMES*, reflecting a pro-inflammatory transcriptional program and expression of genes associated with tissue residency (196).

Beyond intrinsic transcriptional differences, local tissue-derived signals further shape the differentiation and function of these CD8^+^ T cells. Notably, the type 17-like subset, but not the cytotoxic subset, was enriched for a TGF-β-responsive transcriptional signature and exhibited modestly increased expression of *TGFB1* and *TGFBR2*, implicating TGF-β signaling in the differentiation or maintenance of this population (196).

Both cytotoxic and type 17-like subsets upregulated *CXCL13*, a chemokine that could promote B cell recruitment and ectopic lymphoid organization within the synovium (196). In addition, the cytotoxic subset preferentially expressed *CCL5*, which may contribute to recruitment of additional leukocytes into the inflamed synovium (195, 196).

This difference in CD8^+^ T cell composition is consistent with the greater importance of type 17 immunity in psoriatic arthritis and may contribute to the differential efficacy of IL-17A blockade in the two diseases. The identification of these functionally distinct CD8^+^ T-cell programs also has important translational implications (196).

#### Juvenile idiopathic arthritis

Juvenile idiopathic arthritis (JIA) is a chronic autoimmune joint disease in children characterized by persistent synovial inflammation. A study of synovial fluid from patients with JIA identified a population of CD8^+^ T cells expressing PD-1. Compared to CD8^+^ PD-1^−^ cells, CD8^+^ PD-1^+^ cells exhibited increased expression of CD103, CD69, CXCR6, ITGAE, and ITGA1, along with reduced expression of S1PR1. Importantly, despite expressing PD-1, these cells retained strong effector functions, including production of IFN-γ, TNF-α, and granzyme B, and proliferated as indicated by elevated Ki-67 expression (197). Functional evidence for joint-resident CD8^+^ T cells comes from murine antigen-induced arthritis models, where synovial CD8^+^CD44hiCD62LloCD69^+^CD103^+^/^-^ST2^+^ T cells showed genuine residency, and site-specific depletion of these cells during remission attenuated subsequent flare upon antigen re-exposure (194).

A second, transcriptionally distinct CD8^+^ synovial population has been identified in JIA using combined single-cell RNA and T cell receptor sequencing. Unlike the cytotoxic CD103^+^CD69^+^ subset detected in the synovial fluid, this population downregulated EOMES and granzyme expression relative to other synovial CD8^+^ T cells, while upregulating the T_RM_-associated genes CXCR6, ITGA1, and DUSP6. Based on their expression of IL-21 and CXCL13, these cells were proposed to support extrafollicular B cell responses, while TNF and GM-CSF expression suggested potential interactions with myeloid cells. Clonal and trajectory analyses were consistent with recent and repeated antigenic stimulation within the joint. These proposed helper functions, however, were inferred from gene expression rather than functionally validated (198).

Transcriptomic profiling of treatment-naïve JIA synovium demonstrated that synovial tissue T cells, but not their paired synovial fluid or peripheral blood counterparts, upregulated a TGF-β-responsive transcriptional module. Spatial transcriptomics predicted outgoing TGF-β signaling from the myeloid–lymphoid niche harboring these T cells (199), suggesting that tissue-restricted TGF-β exposure may regulate the phenotype of synovial CD8^+^ T cells in JIA, although this has not been functionally tested. Spatial transcriptomics of JIA synovium revealed that CXCR3^+^ CD8^+^ T cells were preferentially colocalized with CXCL9/CXCL10-expressing proinflammatory macrophages, with rare GZMB^+^CD8^+^ T cells contributing high per-cell IFN-γ transcript expression. This finding suggests that there may be signaling between CD8^+^ T cells and inflammatory macrophages. T cell–derived IFN-γ may sustain macrophage proinflammatory polarization while macrophage-derived CXCL9/CXCL10 might recruit additional T cells into the tissue. Whether this interaction creates a functional positive-feedback loop in JIA has not been established (200).

#### Ankylosing spondylitis

Ankylosing spondylitis (AS) is a chronic immune-mediated inflammatory disease of the spondyloarthritis spectrum that primarily affects the spine and sacroiliac joints, causing chronic pain and gradual spinal fusion (ankylosis). Although AS is not generally categorized as a classical autoimmune disease, it is included here because genetic and T cell repertoire studies provide evidence for HLA-associated, antigen-driven CD8^+^ T cell responses.

The association between AS and IBD, including CD and UC is widely recognized. However, while CD8^+^CD103^+^CD49a^+^CXCR6^high^CD62L^-^S1PR1^-^ T cells were enriched in the synovial fluid relative to peripheral blood in AS, this was not observed in patients with RA. These cells produced IL-17A and granzyme B under both resting and stimulated conditions, and additionally secreted IL-10 and TNF-α upon stimulation demonstrating both cytotoxic and Tc17-associated effector features within this T_RM_-like population (201). Qaiyum et al. speculated that the CD8^+^ T_RM_-like population they identified in synovial fluid might originate from the gut (201). Paired gut, synovial tissue, synovial fluid, and peripheral blood samples from AS patients and controls revealed that CD8^+^ T cells expressing the T_RM_-associated markers CD69 and CD103 were increased not only in synovial fluid but also in the gut and peripheral blood of AS patients relative to healthy controls (202). Together, these studies identify phenotypically similar CD8^+^ T cell populations with T_RM_-associated features in the gut and inflamed joints of patients with AS. Whether these populations are developmentally related or traffic between the two sites remains unresolved.

High-throughput TCR repertoire profiling identified expanded CD8^+^ T cell clonotypes associated with HLA-B*27 and HLA-B*38, with patterns consistent with antigen-driven selection. These clonotypes were enriched in synovial fluid and detected at higher frequencies in the peripheral blood of patients with spondyloarthritis than in healthy donors, supporting HLA-restricted antigen recognition (203). Genetic studies also point to non-HLA risk loci that act directly within CD8^+^ T cells to shape their transcriptional and effector programs. RUNX3 harbors an AS-associated regulatory polymorphism (rs4648889) in a promoter-proximal enhancer. In CD8^+^ T cell nuclear extracts, the AS-risk allele showed increased binding of the transcription factor IKZF3, whereas the AS-protective allele showed significantly greater binding of IRF5. Silencing IRF5 in CD8^+^ T cells increased both IFN-γ transcript and protein levels, indicating that IRF5 normally restrains CD8^+^ T cell IFN-γ production (204). These findings provide a direct link between an AS-associated regulatory variant and altered transcriptional control of CD8^+^ T cell effector function.

#### Psoriatic arthritis

Psoriatic arthritis is a chronic immune-mediated inflammatory disease of the musculoskeletal system, characterized by joint inflammation, enthesitis, and, in some cases, axial involvement. Psoriatic arthritis typically develops in individuals with psoriasis, often years after the onset of skin disease, although in some cases joint symptoms may precede cutaneous manifestations. Studies have shown that in the synovial fluid of inflamed joints from patients with psoriatic arthritis, CD8^+^ T cells were present at significantly higher frequencies compared to peripheral blood. These cells exhibited high expression of CD103 and, to a lesser extent, CD69. They also showed elevated expression of CXCR6, ZNF683, and ITGAE, along with downregulation of S1PR1 (69). Approximately 65% of these cells expressed PD-1. Within this population, a subset of CD8^+^ T cells producing IL-17A was identified. Tc17 cells were more frequently found among CD103^+^CD8^+^ T cells and CD69^+^CD8^+^ T cells than CD103^+^CD69^+^CD8^+^ T cells, suggesting that CD103 and CD69 identify functionally distinct CD8^+^ T cell subsets in psoriatic arthritis synovial fluid. Clinically, this IL-17A^+^CD8^+^ population correlated with both disease activity and radiographic joint damage progression, linking this population to structural outcomes rather than inflammation alone (205).

High expression of β7 integrin, which can pair with αE integrin and be co-expressed with CLA, was also observed in the synovial fluid of patients with psoriatic arthritis, raising the possibility of shared or overlapping homing potential between the affected skin and joint compartments (69). In a study comparing peripheral blood T cell subsets between patients with psoriasis and those with subclinical or early psoriatic arthritis, Tc17 cells showed the greatest discriminatory power among all T cell populations examined for identifying which psoriasis patients were transitioning to joint disease (206). In addition, circulating CD8^+^ T cells with a skin T_RM_-associated phenotype were enriched in patients with established psoriatic arthritis compared to those with psoriasis alone (207), and T cell receptor sequencing subsequently identified shared CD8^+^ T cell clonotypes in skin and joint tissue from the same patients, demonstrating clonal overlap between the two compartments (208). Whether these clones traffic directly between skin and joint or arise from a shared circulating pool remains unresolved.

Combined mass cytometry and single-cell RNA sequencing of joint-derived T cells from patients with psoriatic arthritis showed type 17-like CD8^+^CD69^+^CD103^+^ T cells in psoriatic arthritis expressed a type 17-associated transcriptional program with expression of genes associated with tissue residency distinct from the cytotoxic, Eomes-high program that instead predominated in RA synovium (196). The type 17-like subset, but not the cytotoxic subset, was selectively enriched for a TGF-β-responsive transcriptional signature and showed modestly increased expression of TGFB1 and TGFBR2, implicating TGF-β signaling in the differentiation or maintenance of this population (196).

Antigen may drive CD8^+^ T cell expansion within the psoriatic arthritis joint itself. T cell receptor analyses of psoriatic arthritis synovial fluid identified oligoclonal expansions of CD8^+^ T cells, with between 7 and 20 significantly enriched CD8^+^ clones detected per patient, compared to only 1 to 4 CD4^+^ clones. The marked oligoclonal expansion of CD8^+^ relative to CD4^+^ T cells is consistent with selective expansion of particular CD8^+^ clonotypes (209). High-throughput TCR repertoire profiling identified CD8^+^ T cell clonal groups associated with HLA alleles HLA-B*27 and HLA-B*38, with patterns consistent with antigen-driven selection which were enriched in synovial fluid and present at higher frequency in the peripheral blood of patients with spondyloarthritis than in healthy donors. Moreover, several of these clonal groups were shared between patients with ankylosing spondylitis and those with psoriatic arthritis, suggesting a common HLA-restricted, antigen-driven mechanism across the spondyloarthritis spectrum (203). However, nucleotide sequencing of psoriatic arthritis synovial tissue before and during methotrexate treatment found that only a small minority of T cell clones had features suggestive of autoantigen recognition. Thus, the relative contribution of antigen-specific versus bystander-activated CD8^+^ T cells to joint inflammation remains incompletely resolved (210).

Single-cell RNA sequencing of psoriatic arthritis synovium has also shown that memory CD8^+^ T cells are expanded ~3-fold in the joint relative to peripheral blood, with pronounced clonal expansion among cells expressing cycling, activation, and tissue-homing markers (211). CXCR3 was upregulated on synovial CD8^+^ T cells, while its ligands CXCL9 and CXCL10 were increased in psoriatic arthritis synovial fluid. Because these chemokines recruit CXCR3^+^ T cells, this axis may contribute to continued recruitment of CD8^+^ T cells into inflamed joints (211).

### Sjögren’s syndrome

Sjögren’s syndrome (SS) is an autoimmune disease characterized by epithelial inflammation of the salivary and lacrimal glands, leading to dry mouth and dry eyes (212). In a murine model, infiltrating CD69^+^CD103^+/−^ CD8^+^ T cells were the dominant population in the submandibular glands (213). Similarly, CD8^+^CD103^+^CD69^+^ T cells producing granzyme B and IFN-γ have been observed in the salivary glands of SS patients (214). CD8^+^CD103^+^ T cells were selectively expanded in salivary glands compared with controls, with no corresponding expansion in peripheral blood (214). Transcriptomic profiling of SS salivary glands has identified upregulation of ZNF683 (Hobit) and PRDM1 (Blimp-1) relative to controls (214). Tgfb1–3 transcripts were highly expressed within salivary gland tissue, providing a potential local signal for induction of CD103 on infiltration CD8^+^ T cells (213, 215). Whether local metabolic conditions, such as hypoxia or nutrient competition within the chronically inflamed gland, further shape the persistence or function of these CD8^+^ T cells remains largely unexplored, though one transcriptomic study reported a hypoxia-associated gene signature specific to glandular-infiltrating CD8^+^ T cells in SS (216).

Glandular CD8^+^ T cells are heterogeneous. The CD69^+^CD103^-^ subset was clonally linked to circulating T cells, was more cytotoxic, and correlated with disease activity, while CD69^+^CD103^+^ T cells showed no such correlation (217). Notably, Mauro et al. (214) found that CD103^+^ T cells produced significantly more granzyme B and IFN-γ, while Xu et al. did not (217). This discrepancy may reflect methodological differences and underscores that the relative pathogenic contributions of CD103^+^ versus CD103^−^ glandular CD8^+^ T cell populations in SS remain unresolved.

Functional studies in the murine model provide evidence that CD8^+^ T cells and IFN-γ are required for disease development in this model. *Cd8a*-deficient mice were protected from disease, while *Ifng* deficiency reduced CD8^+^ T cell infiltration and glandular damage (213). Therapeutic depletion of CD8^+^ T cells after disease onset also prevented disease manifestations, providing additional evidence for a sustained pathogenic role of these cells. However, these interventions deplete or eliminate circulating as well as resident CD8^+^ T cells and therefore do not distinguish the specific contribution of a non-recirculating resident population from that of circulating CD8^+^ T cells that continuously traffic into the gland.

Recent studies have also linked circulating precursor cells to glandular CD8^+^ T_RM_ cells. Peripheral blood granzyme K-producing CXCR6^+^CD8^+^ T cells were expanded in SS and shared T cell receptor clonotypes with glandular CD69^+^CD103^-^ T cells. Plasma IL-15 was elevated in SS and correlated with the frequency of peripheral granzyme K producing-CXCR6^+^CD8^+^ T cells; IL-15 signaling upregulated CXCR6 and granzyme K on CD8^+^ central memory T cells *in vitro*. The CXCR6 ligand CXCL16 was expressed within CD8^+^ T cell-enriched regions of the gland and was increased in SS. Together, these findings are consistent with a model in which circulating granzyme K^+^CXCR6^+^ are related to the CD69^+^CD103^-^ population, with IL-15 and CXCL16 potentially contributing to their phenotype and recruitment, respectively (217). In a murine model of SS, clonal analyses identified a granzyme K producing-PD-1^+^CD8^+^ subset in the lymph node clonally related to glandular CXCR6^+^CD69^+^CD103^+^ CD8^+^ T cells, and positioned upstream of them by trajectory analysis (218). PD-1-targeted depletion of this population in both the gland and draining lymph node reduced lymphocytic infiltration, inflammatory gene expression, and restored salivary flow and glandular architecture, providing functional evidence for a pathogenic role of this PD-1^+^ CD8^+^ T cell population.

CD8^+^ T cells may cause salivary gland injury through both cytolytic and non-cytolytic mechanisms. Granzyme K producing-CD8^+^ T cells accumulated with disease severity and localized to a seromucous acinar cell population that was selectively depleted in SS. Granzyme K can induce mitochondrial DNA and RNA release and activation of the cGAS-STING-IRF3 pathway leading to type I interferon induced gene expression, without causing caspase-dependent apoptosis. These findings provide a mechanism by which Granzyme K-producing CD8^+^ T cells could contribute to the type I interferon response in SS without inducing apoptotic cell death (219).

**Table 1** summarizes key findings across different autoimmune and immune-mediated inflammatory diseases.

**Table 1:** CD8^+^ tissue-resident memory (T_RM_) T cells in autoimmune and immune-mediated inflammatory diseases.

Disease	Tissue	Markers reported	Type of evidence	Proposed function	Therapeutic relevance	Reference
Vitiligo	Skin	CD49a, CD103, CD69 (lesional CD8^+^ T cells) CXCR3-high CD215/IL-15Rα (keratinocytes); CD122/IL-15Rβ (melanocyte-reactive T_RM_)	Established residency — HLA-A2/tetramer antigen-specificity (human); murine functional/depletion evidence (FTY720 blockade + Thy1.1-antibody depletion of recirculating memory cells, disease reversed despite persisting T_RM_; CXCR3-deficient mice; CXCL10-neutralizing antibody)	Antigen-specific cytotoxic effector against melanocytes; self-amplifying IFN-γ–CXCL9/10–CXCR3 loop drives site-specific relapse after therapy withdrawal	CXCR3/chemokine blockade IL-15/CD122 blockade JAK/STAT inhibition Checkpoint modulation (PD-L1 fusion protein)	(1–14)
Alopecia Areata	Skin	CD103, CD69, ITGAE IL-15/IL-15Rα components	T_RM_-like population — xenograft of lesional T cells into immunodeficient mice reproduces disease (15); selective CD8^+^ T-cell depletion (not CD4^+^, NK, B, γδ) prevents and reverses disease in a murine model (16); though cannot isolate a resident-specific contribution; clonally expanded CD103^+^CD69^+^ T cells persist in lesional skin after treatment withdrawal	Antigen-specific (trichohyalin/TRP2) cytotoxic effector collapsing hair-follicle immune privilege via IFN-γ; drives relapse at the same follicles	JAK/STAT inhibition	(17–21)
Systemic Sclerosis	Skin	CD69, CCR10 (largely CD103-negative) CCR4, CLA PD-1-high/CD28-low (exhausted, metabolically impaired)	T_RM_-like population — human blood/lesional-skin marker and functional profiling; a distinct “T migratory memory” subset with mixed residency/recirculation features identified, arguing against a uniform resident population (22), scRNA-seq/epigenomic profiling identifies two developmentally linked subsets, with the early subset directly inducing tissue damage and fibrosis in ex vivo skin explant assays (23)	Profibrotic cytotoxic effector (granzyme B, IFN-γ, IL-13) linked to skin fibrosis; early KLRB1^+^IL7R^+^ cytolytic/Tc2-Tc17 subset predominates in early diffuse disease and drives fibrosis, transitioning to a late KLRG1^+^IL7R^−^ exhausted subset	None specifically evidenced	(24, 25)
Systemic Sclerosis	Lung (SSc-ILD)	CD69, ZNF683 (HOBIT), ITGAE S1PR1-low	T_RM_-like population — single-study human tissue transcriptomic/marker characterization (SSc-ILD lung vs. healthy lung); expanded proportion of CD8^+^ T_RM_	trajectory analysis suggests that resting CD8^+^ T cells progressively differentiate into CD8^+^ effector memory and then CD8^+^ T_RM_ cells	None specifically evidenced	(26, 27)
Cutaneous Lupus Erythematosus	Skin	CD103, CD69 CCR7 (antimalarial-resistant subset)	T_RM_-like population — conflicting reports on marker–severity correlation (28, 29)	IFN-driven (JAK/STAT, CXCL9/10/11–CXCR3) cytotoxic effector; contested role in antimalarial resistance and IL-17 production	JAK/STAT inhibition IFN-γ blockade (AMG811) — reduced CXCL10	(30)
Psoriasis	Skin	CD103, CD49a, CD69 CCR6, IL-23R (IL-17-poised subset)	Established residency — xenotransplant of non-lesional skin induces psoriasis in immunodeficient mice, prevented by pre-transplant CD8^+^ depletion (31, 32); anti-E-selectin blockade of new T-cell recruitment failed clinically, supporting a resident rather than recruited driver (33); clonally restricted, antigen-specific (ADAMTSL5/HLA-C*06:02) T_RM_ persist through clinical resolution	IL-17-producing antigen-specific effector persisting through treatment	IL-17A blockade (secukinumab) IL-23 blockade (guselkumab) S1PR1 modulation (fingolimod, ponesimod) IL-15/CD122 blockade JAK/STAT inhibition	(4, 34–44)
Inflammatory Bowel Disease	Intestine	CD69, CD103, KLRG1 (mutually exclusive subsets) CXCR6, Hobit, Blimp-1; low S1PR1/KLF2	T_RM_-like population —some studies report contraction of the T_RM_ compartment in active disease (45), others report expansion/reprogramming of pathogenic subsets (46, 47); — not yet resolved	Persistent clonally restricted CD8^+^ T cells predict post-surgical recurrence in Crohn's disease	S1PR1/egress modulation JAK/STAT inhibition	(48–54)
Autoimmune Hepatitis	Hepatobiliary system	CD69, CD103 (CD103-dependent anchoring appears human-specific, murine hepatic T_RM_ instead rely on LFA-1-mediated sinusoidal patrolling), PRDM1 (BLIMP1), CXCR3, PD-1, HOBIT (upstream repressed by CIC–ETV5 axis)	T_RM_-like population — PRDM1/BLIMP1 and CCL5–CCR5 signature identified in peripheral blood CD8^+^ T cells and cross-validated against liver transcriptomes (55); antigen specificity shown via HLA-A2 tetramer (CYP2D6); but not yet linked directly to the hepatic T_RM_ population itself	Cytotoxic effector (IFN-γ, TNF-α, granzyme B) anchored via CD103/E-cadherin; CCL5–CCR5 self-amplifying loop with NK cells, persistence supported by autocrine IL-2 signaling and hepatocyte-derived IL-7, in addition to the IL-15/TGF-β signals	None specifically evidenced	(55–63)
Autoimmune Cholangitis	Hepatobiliary system	CD69, CD103, CD62L-negative Cxcr6, Prf1, Gzma, Runx3, Hobit; S1pr1-low	Established residency — murine parabiosis functionally confirmed non-circulating residency; genetic/pharmacological NUDT1 blockade eliminated the T_RM_ population and alleviated disease (causal); human PDC-E2-specific CD8^+^ T cells enriched ~10-fold in liver vs. blood by HLA-A2 tetramer	Directly cytotoxic to bile duct epithelium; NUDT1/PARP1/TGFβ-Smad-dependent survival program sustains residency and portal fibrosis	PD-1-directed CAR-T depletion	(64, 65)
Primary Sclerosing Cholangitis	Hepatobiliary system	CD69, CD103, CD49a CXCR6, CCR6, α4β7, CCR9, CXCR3	T_RM_-like population — transgenic shared-antigen model shows gut-primed CD8^+^ T cells are necessary for antigen-dependent cholangitis, the experiments track effector kinetics over approximately one week rather than long-term persistence; mice lacking the shared antigen in bile ducts fully protected despite equivalent gut priming (66); human biliary CD8^+^ T cells are characterized by marker co-expression (CD69/CD103/CD49a) alone, without direct evidence of non-recirculating residency	Gut-primed effector migrating to liver via the gut–liver axis; forms local network with neutrophils (CXCL8/CXCL16)	None specifically evidenced	(67)
Diabetes (Type 1)	Pancreas	CD103 (acquired over time), CD69, FABP4 (metabolic marker, murine)	T_RM_-like population — human islet marker co-expression; explicitly caveated in the source studies: similar T_RM_-like phenotypes also found in healthy pancreas, so presence alone does not establish pathogenicity; Murine NOD model now provides T_RM_-directed depletion evidence (anti-CD69 antibody delays onset and reduces incidence, phenocopying FABP4 knockout)	Diabetogenic potential tracks with CXCL10-driven recruitment more than direct cytotoxicity; produces IFN-γ, IL-22, IL-15, CXCL9, FABP4-dependent fatty acid oxidation sustains CD8^+^ T_RM_ survival in the islet; FABP4 drives CXCL10 secretion, identifying CD8^+^ T_RM_ cells as a direct cellular source of the CXCL10 signal that recruits circulating CD8^+^ T cells (murine)	IL-17A/F blockade S1PR1 modulation (fingolimod + sitagliptin) FABP4 inhibition (proposed, not yet tested)	(68–76)
Lupus Nephritis	Renal	CD69, CD103 Granzyme B, perforin	T_RM_-like population — murine antigen-driven renal T_RM_ differentiation; persistent T-cell clonotypes detected across serial human biopsies; single-cell transcriptomics reveals substantial heterogeneity (multiple PD-1^+^ subsets); no depletion/functional causality study specific to renal _TRM_ described	Cytotoxic effector shaping local humoral autoimmunity via crosstalk with atypical B cells in tertiary lymphoid structures	IFN-γ blockade (AMG811) S1PR1 modulation CD40-CD40L blockade (dapirolizumab pegol) JAK/STAT inhibition (tofacitinib)	(30, 43, 77–87)
Multiple Sclerosis	Brain (CNS)	CD44, CD69, CD49a, PD-1 (mixed CD103^+/-^) CXCR6, Ki-67; S1PR1/CCR7-negative; CD20-dim (subset)	Established residency (causal, murine) — transgenic neo-self-antigen reactivation model shows quiescent T_RM_ converted to pathogenic effectors upon antigen re-exposure, full effector differentiation dependent on CD4^+^ T-cell help; conflicting marker reports across studies (CD103 without CD69, CD69 without CD103)	Antigen-reactivation-driven effector engaging microglia (CXCL16–CXCR6, CCL5–CCR1) to sustain demyelination; IL-33/ST2 signaling on oligodendrocytes drives TCF-1-high precursor-to-pathogenic-T_RM_ transition	IL-33/ST2 blockade CD40-CD40L blockade (frexalimab) CD4+ help withdrawal (established T_RM_) CD20 depletion (rituximab)	(88–97)
Rheumatoid Arthritis	Joints	CD44-high, CD62L-low, CD69, CD103^+/-^, ST2 CXCR6, CD49a, PD-1; low S1PR1/KLF2; granzyme B/perforin-high	Established residency — murine site-specific depletion during remission attenuates subsequent flare (causal); cells resist FTY720-induced egress, fail to migrate toward CCL21, persist over 8 months without inter-joint migration (98); human synovial fluid marker/functional characterization resolves cytotoxic subsets	Cytotoxic subset drives perforin-mediated histone citrullination/NET formation linked to ACPA generation;	IL-17A blockade shows limited efficacy (contrasts with psoriatic arthritis)	(99, 100)
Juvenile Idiopathic Arthritis	Joints	PD-1, CD103, CD69 CXCR6, ITGAE, ITGA1; low S1PR1; separate subset: CD40LG, IL21, CXCL13, TOX, ZNF683 (HOBIT)	T_RM_-like/established mixed — human synovial marker/functional correlates interpreted alongside murine functional-residency evidence from an RA model (98); a proposed helper-like population is inferred from gene expression only, not functionally validated; a HOBIT^+^ population is defined by marker expression alone, without functional confirmation of residency	Cytotoxic effector (IFN-γ, TNF-α, granzyme B) plus a proposed peripheral-helper-like population supporting B-cell/myeloid activation via IL-21/CXCL13/TNF/GM-CSF rather than direct cytotoxicity	None specifically evidenced	(101–104)
Sjögren's Syndrome	Salivary glands	CD69, CD103 (two functionally distinct subsets) CXCR3, CXCR6; Hobit, Blimp-1; granzyme K/B	T_RM_-like population — CD8a or IFN-γ knockout prevents disease; therapeutic CD8^+^ depletion protects even after disease onset (105); though cannot isolate a resident-specific contribution; human data conflict on whether the CD103^+^ or CD103^-^ subset is more pathogenic (106, 107); PD-1-targeted depletion of a precursor-to-effector axis reduces inflammation and restores glandular function (108)	IFN-γ/CXCL9/CXCL10/CXCR3 self-amplifying loop; granzyme K drives non-apoptotic epithelial injury via cGAS-STING signaling	PD-1-targeted depletion (precursor-to-effector axis) CD103-directed depletion (SAP-103) CXCR3/chemokine blockade	(109–111)
Ankylosing Spondylitis	Joints	CD103, CD49a, CXCR6-high CD62L/S1PR1-negative; PD-1, TIGIT, LAG-3 (exhaustion-resistant)	T_RM_-like population — human synovial fluid/gut/blood marker characterization across paired compartments (112); genetic evidence links an AS-risk RUNX3 enhancer polymorphism to altered IRF5 binding and CD8^+^ IFN-γ regulation (113)	Constitutively IL-17A/granzyme B-producing Tc17-like effector; gut-joint recirculation proposed as mechanistic link to IBD association	JAK/STAT inhibition	(43, 114, 115)
Psoriatic Arthritis	Joints	CD103, CD69 CXCR6/CXCL16, ZNF683 (HOBIT), ITGAE; low S1PR1; PD-1 (~65%)	T_RM_-like population — human synovial fluid marker/functional (IL-17A production) characterization; shared CD8^+^ clones identified between skin and joint tissue in the same patients (116), supporting a skin–joint recirculation axis	Type-17-like effector (RORA, AHR, BATF, ZNF683) distinct from RA's cytotoxic program, offering a mechanistic explanation for differential IL-17A blockade efficacy between the two diseases, TGF-β-responsive transcriptional signature	IL-17A blockade effective (contrasts with RA) JAK/STAT inhibition	(99, 117–120)

Established residency: supported by functional evidence (e.g., parabiosis, FTY720/antibody-mediated blockade of recirculating cells, genetic or antibody-mediated depletion, or antigen-reactivation studies) demonstrating that a locally persistent, non-recirculating CD8+ T-cell population is required for or sufficient to drive disease.

T_RM_-like population: identified primarily by surface marker co-expression (e.g., CD69, CD103, CD49a) without direct functional confirmation of non-recirculating residency.

## Discussion

### Targeting T_RM_ cells: therapeutic strategies

In the preceding sections, we have outlined the role of CD8^+^ T_RM_ cells as long-lived immune populations that persist within peripheral tissues, strategically positioned to provide rapid protection. However, increasing evidence indicates that T_RM_ cells can acquire pathogenic functions, contributing to chronic inflammation. Given this dual role, an important question arises: can CD8^+^ T_RM_ cells be therapeutically targeted to mitigate autoimmune pathology? More specifically, is it possible to intervene at different stages of CD8^+^ T_RM_ cell biology in order to control disease progression?

**Figure 2:**
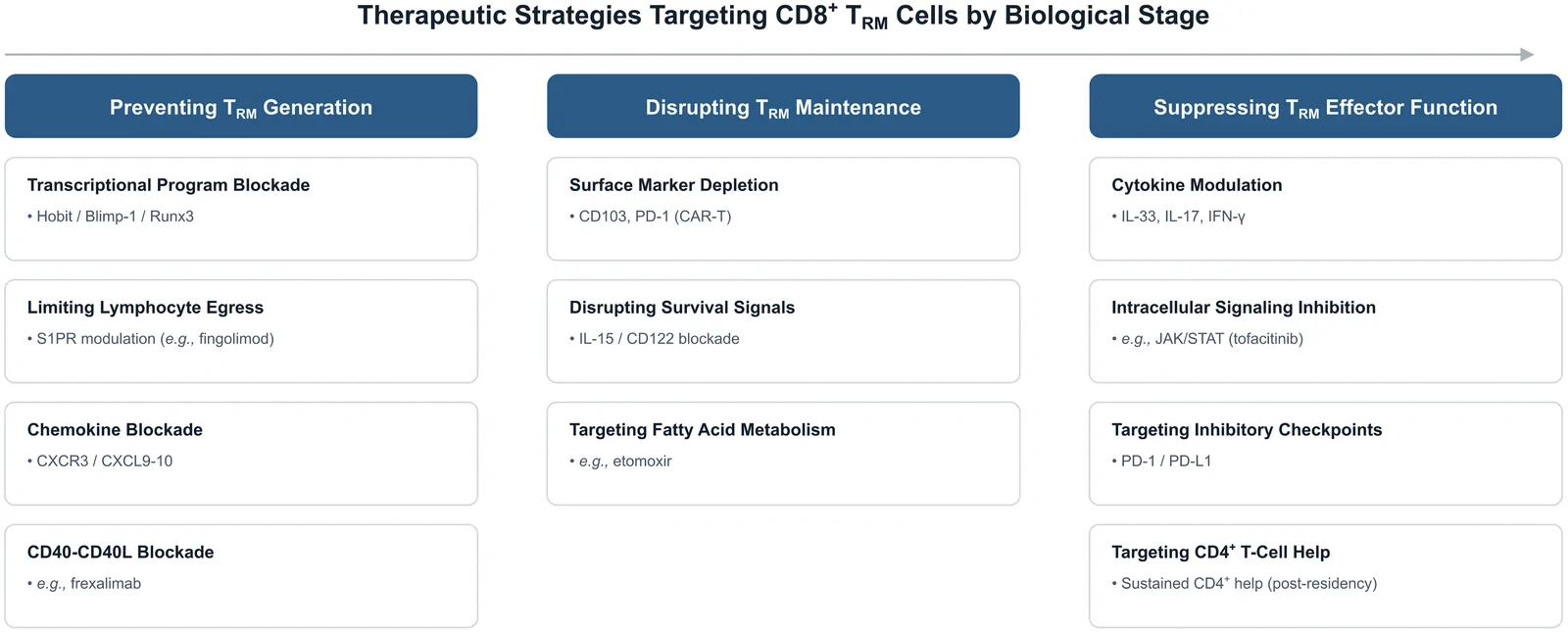
Strategies to prevent TRM generation include blockade of the core transcriptional program, limiting lymphocyte egress, chemokine blockade, and CD40-CD40L blockade. Strategies to disrupt TRM maintenance include depletion of key surface markers, disruption of survival signaling, and targeting of fatty acid metabolism. Strategies to suppress TRM effector function include cytokine modulation, inhibition of intracellular signaling, targeting inhibitory checkpoints, and targeting of sustained CD4^+^ T-cell help.

In this context, potential therapeutic strategies are organized according to the stage of CD8^+^ T_RM_ cell biology they primarily affect: tissue entry and establishment, long-term maintenance, and effector function (**Figure 2**). Within each stage, we distinguish interventions that primarily target T_RM_-associated pathways from those that more broadly modulate the immune response.

### Preventing T_RM_ generation and tissue establishment

#### A. Targeting transcriptional programs

Transcription factors, including Hobit, Blimp-1, and Runx3 contribute to the establishment and maintenance of CD8^+^ T_RM_ cell differentiation programs, although their relative importance varies across tissues and inflammatory contexts. In principle, disrupting these transcriptional programs could limit the formation and persistence of pathogenic CD8^+^ T_RM_ cells. However, direct therapeutic targeting of these factors remains challenging. First, unlike cell surface proteins, transcription factors are not readily accessible to antibody-based therapies. Second, their expression often spans multiple cell types and biological contexts, which increases the risk of significant off-target effects. Third, transcriptional networks are highly redundant; inhibition of a single factor may be compensated by alternative pathways, limiting therapeutic efficacy. In addition, the requirement for specific transcription factors varies across tissues, further complicating the development of broadly applicable strategies. As such, targeting transcriptional programs remains largely conceptual rather than a tested therapeutic avenue.

#### B. Limiting lymphocyte egress

S1PR1 modulators bind to S1PR1 and promote its internalization, thereby limiting responsiveness to S1P egress signals. This limits lymphocyte egress from secondary lymphoid organs, reducing the pool of circulating lymphocytes available for recruitment into inflamed tissues. Some tissue-resident populations can give rise to cells that reenter the circulation under inflammatory conditions; S1PR1 modulation could potentially limit subsequent redistribution of these cells and prevent disease dissemination. Thus, S1PR1 modulators primarily act by altering systemic lymphocyte trafficking rather than directly targeting T_RM_-specific pathways. Nevertheless, by limiting the recruitment of circulating T cells into inflamed tissues, S1PR1 modulators may indirectly reduce the establishment of new pathogenic CD8^+^ T_RM_ cell populations.

Currently, four S1PR modulators are approved for MS and, despite differences in receptor selectivity, all inhibit lymphocyte egress from secondary lymphoid organs (220, 221). S1PR modulators have also shown efficacy in IBD, reducing intestinal inflammation and pro-inflammatory cytokines in colitis models (222, 223) and improving disease control in patients with UC and CD (224–226). In experimental models of SLE, fingolimod extended survival (227, 228), while also decreasing infiltration by CD4^+^ and CD8^+^ T lymphocytes (229). In addition, fingolimod attenuated epidermal hyperproliferation in a murine model of psoriasis (230). Similarly, in a phase II clinical trial, oral Ponesimod demonstrated clinical benefit (231). In diabetic mouse models, combination treatment with fingolimod and sitagliptin produced marked improvement (232). While S1PR1 modulation has demonstrated promising therapeutic benefits across several autoimmune conditions, important limitations remain, particularly regarding systemic immunosuppression and potential off-target effects (221, 233–236).

#### C. Targeting chemokine pathways

Chemokine pathways regulate recruitment and positioning of CD8^+^ T cells within tissues and can therefore influence both CD8^+^ T_RM_ cell establishment and interactions with local immune and stromal cells. Targeting chemokines and their receptors may therefore disrupt both the recruitment of CD8^+^ T_RM_ precursors into tissues and the niches that support the maintenance and effector function of established CD8^+^ T_RM_ cells.

In a mouse model of Sjögren’s syndrome, blockade of CXCR3 using a neutralizing antibody reduced CD8^+^ T cell infiltration into tissues and prevented disease development (237). Similarly, in murine models of vitiligo, targeting the CXCR3/CXCL10 axis prevented depigmentation and even induced repigmentation, leading to disease reversal (98). However, chemokine dependence may differ between tissue entry and intratissue migration. Salivary gland CD8^+^ T_RM_ cells, for example, can exhibit largely Gαi-independent intratissue motility (238, 239). Moreover, CXCR3 and its ligands are not CD8^+^ T_RM_ cell-restricted but are broadly expressed by activated CD4^+^ and CD8^+^ T cells as well as NK cells. Consequently, CXCR3 blockade influences lymphocyte trafficking well beyond the T_RM_ compartment.

#### D. Disrupting CD40-CD40L signaling

In settings in which pathogenic CD8^+^ T_RM_ cell response requires CD4^+^ T cell help, disruption of CD40-CD40L signaling may provide an indirect strategy for limiting T_RM_ effector differentiation. CD40-CD40L interactions are a major mechanism by which CD4^+^ T cells license antigen presenting cells to support CD8^+^ T cell responses. Clinical studies targeting this pathway have shown efficacy in autoimmune disease: frexalimab, an Fc-engineered anti-CD40L antibody, reduced brain lesions by 79–89% versus placebo in a phase 2 trial in relapsing MS (240); dapirolizumab pegol, a PEGylated anti-CD40L Fab’ fragment, met its primary endpoint in SLE (241). Neither trial was designed to assess effects on CD8^+^ T_RM_ cells specifically. Vincenti et al. (76) added an important caveat: they speculated that therapies blocking circulating CD4^+^ T-cell entry into the CNS could interfere with CD8^+^ T_RM_ cell pathogenicity early in disease, whereas established CD8^+^ T_RM_ populations may become less dependent on newly recruited CD4^+^ T cell help during chronic disease. If this model applies to human disease, interruption of CD40-CD40L signaling may be most effective before the establishment of chronic, compartmentalized CD8^+^ T_RM_ cell responses.

### Disrupting CD8^+^ T_RM_ cell maintenance and persistence

#### A. Surface-marker-directed depletion of established CD8^+^ T_RM_ cells

One strategy for eliminating established pathogenic CD8^+^ T_RM_ cells is to target surface molecules enriched on these populations. For example, recent studies have depleted CD103-expressing cells in the oral mucosa using a toxin-conjugated anti-CD103 antibody (SAP-103), resulting in loss of the targeted CD103^+^ population (215). In a murine model of Sjögren’s syndrome, local CD103 blockade within the glandular tissue attenuated tissue damage and improved salivary secretion (214). However, this strategy has important limitations. First, the expression of surface markers is not uniform across tissues and disease states; second, expression of some markers may not be restricted to pathogenic CD8^+^ T_RM_ cells (170). As a result, using these receptors for depletion may result in off-target effects.

Another depletion strategy may involve the CD20^dim^ T cell population found in MS. Anti-CD20 monoclonal antibodies, widely used in MS therapy, eliminate CD20-expressing B cells and concurrently reduce peripheral CD8^+^ T cells with low-level CD20 expression (242). In human CD20-transgenic mouse models of MS, the efficacy of rituximab was greatest when treatment induced substantial T-cell depletion (243). While this study did not specifically demonstrate depletion of CD8^+^ T cells, a separate study demonstrated that rituximab depleted circulating CD20^+^ T cells in the peripheral blood of patients with MS, with approximately 45% of CD20^+^ T cells co-expressing CD8 and 55% co-expressing CD4 (190). In mouse models of MS, rituximab treatment significantly reduced CD19^+^ B cells, and notably, also decreased CD3^+^ T cell accumulation within the brain. This treatment reversed demyelination to near-normal levels (244). These findings raise the possibility that depletion of CD20-expressing T cells contributes to the therapeutic effects of anti-CD20 treatment. However, whether anti-CD20 therapy directly depletes established CD20^dim^ CD8^+^ T_RM_ cells within human CNS lesions has not been established.

A third strategy has used PD-1 expression to target a pathogenic CD8^+^ T cell population with Chimeric Antigen Receptor T-cells (CAR-T) in experimental cholangitis. This approach effectively reduced inflammation in the portal tracts, biliary injury, and fibrotic remodeling in a mouse model of cholangitis (155). This approach was based on the observation that CD8^+^ T_RM_ cells are characterized by elevated PD-1 expression, enabling their recognition and cytotoxic targeting. In this model, there was a selective reduction of CD8^+^ T_RM_ cells, with minimal impact on other CD8^+^ T cell populations. However, because PD-1 is also expressed by activated and chronically stimulated T cells, the specificity of this approach depends on the cellular context and level of PD-1 expression. Given that CD8^+^ T_RM_ cells are distributed across multiple organs, CAR-T–mediated targeting may lead to unintended depletion of T_RM_ populations in non-target tissues, warranting caution in clinical translation.

#### B. Disrupting survival signals

In murine models of vitiligo, an anti-CD122 antibody targeting the shared IL-2/IL-15 receptor β chain led to reduced disease progression and decreased melanocyte loss (91). Additionally, this therapy reduced IFN-γ levels within two weeks and resulted in a decline in pathogenic CD8^+^ T_RM_ cells after eight weeks of treatment (91). Furthermore, blockade of IL-15 reduced CD8^+^ T-cell infiltration in a human psoriasis xenograft model (245). However, IL-15 inhibition does not only affect CD8^+^ T_RM_ cells; CD4^+^ and CD8^+^ effector memory T cells, as well as NK cells, are also rapidly reduced (246), raising concerns about the clinical utility of this approach. Redundancy is also a factor: IL-7 and possibly IL-2 can partially compensate for IL-15 loss. Whether this redundancy limits the durability of IL-15-directed therapy in pathogenic CD8^+^ T_RM_ cells remains unclear.

### Targeting metabolic pathways

In several tissues, CD8^+^ T_RM_ cells depend on uptake and oxidation of exogenous FFAs for long-term survival. The administration of pharmacologic agents that block lipid metabolism (etomoxir or trimetazidine) reduced the survival of these cells (77, 247). However, metabolic targeting of CD8^+^ T_RM_ cells may not be a straightforward strategy. FABPs encompass an expanded group of isoforms, with distinct members regulated in an environment-dependent manner and according to local fatty acid availability. These distinct patterns of FABP expression are driven by local microenvironmental cues, and with changes in FABP expression patterns, CD8^+^ T cells can adapt to different host tissues. Notably, selective FABP isoform expression is also observed in other immune cells, for example, tissue-resident NK cells. Inhibition of fatty-acid uptake or oxidation may affect protective tissue-resident immune populations in addition to pathogenic CD8^+^ T_RM_ cells.

### Suppressing CD8^+^ T_RM_ cell effector function

#### A. Cytokine modulation

IL-33 levels in both the periphery and the CNS are increased in MS. Intracerebral administration of an AAV-based ST2-Fc decoy receptor, designed to neutralize IL-33 signaling, substantially reduced accumulation of CD8^+^ T cells in the brain and ameliorated the clinical course of disease. This effect was restricted to the central nervous system, with no observable changes in the spleen, reinforcing the local action of IL-33 in CD8^+^ T_RM_ cell regulation (191). These findings support a local role for IL-33 in promoting CD8^+^ T cell accumulation and persistence within the CNS and identify the IL-33/ST2 axis as a potential therapeutic target for limiting pathogenic CD8^+^ T_RM_ cell responses in MS (191).

Serum concentrations of CXCL10 in patients with SLE were significantly reduced following treatment with AMG 811 (a human anti–interferon-γ antibody) (249). However, the monoclonal antibody AMG 811 has shown only limited and transient effects in lupus nephritis (250).

In an experimental model of T1D, neutralization of either IL-17A or IL-17F prevented diabetes induction by pathogenic Tc17 cells (248).

Secukinumab, an IL-17A inhibitor used in psoriasis, has demonstrated clinical benefits (251). However, TCRαβ^+^ T cells persist in previously affected sites even after treatment, including in skin that appears clinically normal. These cells retain the ability to produce IL-17 and IFN-γ and may contribute to the reactivation of psoriatic lesions (133, 252, 253). The GUIDE clinical trial has shown that early intervention with the IL-23 inhibitor guselkumab was associated with a rapid reduction in CD8^+^ T_RM_ cells toward levels observed in unaffected skin and an increase in IL-10-producing Tregs (254, 255).

There are several important limitations associated with targeting interleukins as therapeutic strategies. Cytokine redundancy may limit efficacy. For instance, in psoriasis, blockade of IL-23 does not fully suppress inflammation, as IL-1β can still induce IL-17 production by T cells. In addition, dependence on specific interleukins varies across different tissues. More importantly, none of the cytokines discussed above are CD8^+^ T_RM_-specific signals, so blockade of any of them reaches well beyond the CD8^+^ T_RM_ compartment. Taken together, these considerations highlight the need for careful evaluation of tissue-specific effects and broader immunological consequences when designing cytokine-targeted therapies.

#### B. Targeting signaling pathways

Intracellular signaling pathways have been implicated in regulating aspects of CD8^+^ T_RM_ cell maintenance and function. The JAK/STAT pathway is a principal downstream signaling cascade of cytokine receptors and plays a central role in shaping CD8^+^ T_RM_ cell responses, particularly through interferons and common γ-chain cytokines (256, 257). Treatment with tofacitinib, a first-generation JAK inhibitor, resulted in amelioration of disease activity, including nephritis and dermatitis, in lupus-prone MRL/lpr mice, along with a significant reduction in CD8^+^ T cells (258). Pharmacologic inhibition of the JAK/STAT pathway (e.g., tofacitinib) has demonstrated clinical efficacy in multiple autoimmune conditions such as psoriasis, psoriatic arthritis, UC, CD, and AS (259).

Although JAK inhibitors can produce rapid clinical improvement in CLE, vitiligo, and AA, lesions frequently recur after treatment withdrawal. This recurrence is consistent with persistence of local disease memory and suggests that JAK inhibition may suppress CD8^+^ T_RM_ IFN-γ signaling and prevent new T cell accumulation without permanently eliminating the pathogenic tissue-resident population (260). Upon re-initiation of treatment, lesions improved again in CLE. JAK inhibition also reduced CXCL10 levels in both CLE and vitiligo, resulting in impaired recruitment of circulating CD8^+^ T cells into the skin (100, 261–264).

Overall, this pathway is highly interconnected and broadly active across immune and non-immune cell types, so its modulation is expected to produce widespread, context-dependent effects beyond CD8^+^ T_RM_ cells.

#### C. Targeting inhibitory checkpoint pathways

Immune checkpoint pathways provide an alternative strategy for suppressing pathogenic CD8^+^ T_RM_-cell activity. PD-1 is an inhibitory receptor induced following T cell activation and is often expressed on chronically stimulated and exhausted T cells. It is also expressed by many CD8^+^ T_RM_ cells (21). PD-1 was required for the local expansion of antigen-specific CD8^+^ T cells with a tolerant phenotype in the skin, by limiting their effector functions and restricting their interaction with APCs. Conversely, pharmacological inhibition of PD-1 promoted cutaneous inflammation and was associated with the development of lichenoid-like skin lesions (265). A recent study suggested that a PD-L1 fusion protein decreased melanocyte-reactive CD8^+^ T cells in vitiligo lesions, increased Tregs in the skin, and successfully reversed depigmentation in a murine model of vitiligo after six weeks of treatment. Although the therapeutic effect was relatively durable, it was not permanent (266). Importantly, the therapeutic consequence of targeting PD-1 depends on the mode of intervention. Whereas enhancing PD-1 signaling suppresses pathogenic T_RM_-cell activity, PD-1 can also be exploited as a surface marker for selective CAR-T-mediated depletion of established T_RM_ cells.

#### D. Targeting CD4^+^ T- cell help to established CD8^+^ T_RM_ cells

The requirement for continued CD4^+^ T cell help after CD8^+^ T_RM_ cell establishment remains less clear. Mockus et al. (72) showed that CD4^+^ T cell depletion after brain CD8^+^ T_RM_ cell establishment did not reduce CD8^+^ T_RM_ cell numbers, but decreased the CD103^+^ fraction, made them dependent on replenishment by circulating T cells, and impaired recall IFN-γ production, indicating that CD4^+^ help remained necessary for CD8^+^ T_RM_ cell function even after residency was established. In contrast, Vincenti et al. proposed that CD8^+^ T_RM_ cell responses may become progressively less dependent on newly recruited CD4^+^ T cell help during chronic disease (76). If established T_RM_ cells were CD4-independent, ongoing CD4 depletion would be expected to have little functional effect. However, Mockus et al. (72) demonstrated a measurable loss of residency identity and recall capacity following chronic CD4 depletion. Differences in model, disease stage, and duration of antigen exposure may contribute to these apparently divergent findings.

**Table 2** summarizes the therapeutic strategies discussed above.

**Table 2:** Therapeutic strategies influencing pathogenic CD8^+^ T_RM_ cells, organized by biological stage.

Strategy	Key biological insight	Reference
Preventing CD8^+^ T_RM_ Generation and Tissue Establishment		
Transcriptional Program Blockade Hobit, Blimp-1, Runx3	Targets transcription factors that drive CD8^+^ T cells into the CD8^+^ T_RM_ differentiation program upstream of tissue entry. Strong genetic evidence from murine knockout/overexpression models, but no therapeutic validation in humans; these factors are intracellular, pleiotropic across cell types, and functionally redundant, limiting druggability.	(1–3)
Limiting Lymphocyte Egress S1PR1 modulation (fingolimod, siponimod, ozanimod, ponesimod, cenerimod)	Targets S1PR1-dependent egress from lymphoid organs, blocking recruitment of CD8^+^ T_RM_ precursors. Reduces recirculation-driven disease activity across multiple tissues (CNS, gut, kidney, skin, pancreas). Established CD8^+^ T_RM_ are largely unaffected. Not CD8^+^ T_RM_-specific: acts on systemic lymphocyte trafficking broadly, with associated CNS-penetration and cardiovascular risk.	(4–22)
Chemokine Blockade CXCR3 / CXCL9-10	Targets CXCR3-dependent recruitment and intra-tissue positioning of CD8^+^ T_RM_ precursors; CXCL9 governs recruitment while CXCL10 governs positioning and effector function. Not CD8^+^ T_RM_-restricted (broadly expressed on activated T and NK cells) and dispensable in tissues where CD8^+^ T_RM_ migrate via adhesion-based, chemokine-independent motility. Required for initial tissue entry but becomes dispensable for CD8^+^ T_RM_ motility once cells are established.	(23–29)
CD40-CD40L Blockade e.g., frexalimab, dapirolizumab pegol	Targets CD4^+^ T-cell licensing of dendritic cells, the upstream step through which CD4^+^ help drives CD8^+^ T_RM_ effector differentiation. Likely a timing-dependent effect: efficacy may decline once established CD8^+^ T_RM_ become CD4-independent with chronicity.	(30–34)
Disrupting CD8^+^ T_RM_ Maintenance and Persistence		
Surface-Marker-Directed Depletion anti-CD103 (SAP-103)	Directly ablates CD103-expressing CD8^+^ T_RM_ already established in tissue. Selectively induces apoptosis in the resident population while sparing circulating T cells. CD103 expression is tissue-variable (high in epidermis, low in liver/brain), and not CD8^+^ T_RM_-exclusive, risking off-target depletion.	(35–42)
CD20-Directed Depletion rituximab	Targets a CD20-dim subset among CD8^+^ T_RM_ cells. Effect on CD8^+^ T_RM_ is likely indirect/secondary to B-cell depletion in most models, and only a minority of CD20^+^ T cells are CD8^+^.	(43–46)
PD-1-Directed Depletion CAR-T	Exploits elevated PD-1 expression on established CD8^+^ T_RM_ as a CAR-T-mediated depletion. Minimal impact on other CD8^+^ subsets. CD8^+^ T_RM_ cells are distributed across multiple organs, so on-target/off-tissue depletion in non-diseased sites remains an unresolved risk.	(47)
Disrupting Survival Signals IL-15 / CD122 blockade	Targets IL-15/CD122 signaling required for CD8^+^ T_RM_ survival after establishment. Not CD8^+^ T_RM_-specific: also depletes CD4+/CD8+ effector-memory and NK cells; efficacy is likely tissue-dependent, and IL-7/IL-2 redundancy limits durability of CD8^+^ T_RM_ suppression from IL-15 blockade alone.	(48–51)
Targeting Fatty Acid Metabolism e.g., etomoxir, trimetazidine	Targets exogenous free-fatty-acid uptake and oxidation, a metabolic dependency of CD8^+^ T_RM_. Pharmacologic blockade reduces CD8^+^ T_RM_ survival in vitro. FABP4 knockout mimics CD8^+^ T_RM_ depletion. FABP isoform usage varies by tissue and is shared with other resident populations (e.g., tissue NK cells).	(42, 52–54)
Suppressing CD8^+^ T_RM_ Effector Function		
IL-33 / ST2 Blockade	Targets oligodendrocyte-derived IL-33 signaling through ST2 on CD8^+^ T cells, which drives the transition from a TCF-1(high) stem-like precursor to a pathogenic T_RM_ state. CNS-restricted blockade reduces CD8^+^ T_RM_ accumulation without affecting the spleen. Positions IL-33/ST2 as a tissue-local checkpoint on CD8^+^ T_RM_ differentiation rather than a pan-T_RM_ target.	(55)
IL-17A / F Blockade	Targets IL-17A and IL-17F, both required for Tc17-driven pathology. Suppresses inflammatory output of the CD8^+^ T_RM_ without necessarily depleting the resident cells. CD8^+^ T_RM_ persisting after treatment retain the capacity to produce IL-17 and IFN-γ.	(56–60)
IL-23 Blockade e.g., guselkumab	Targets IL-23 signaling upstream of the IL-17-producing CD8^+^ T_RM_ subset. Early intervention rapidly reduces CD8^+^ T_RM_ toward baseline levels and increases IL-10^+^ Tregs. IL-1β can sustain IL-17 production independent of IL-23, so blockade does not fully suppress the pathway.	(61, 62)
IFN-γ Blockade AMG811	Targets CD8^+^ T_RM_-derived IFN-γ. Reduces downstream CXCL10 expression. Only transient clinical benefit; IFN-γ blockade alone is insufficient to durably suppress CD8^+^ T_RM_-driven inflammation.	(63, 64)
Intracellular Signaling Inhibition JAK/STAT (tofacitinib)	Suppresses cytokine-dependent activation of established CD8^+^ T_RM_ but does not eliminate the resident cells. Highly pleiotropic: effects extend well beyond the CD8^+^ T_RM_ compartment.	(65–71)
Inhibitory Checkpoint Modulation PD-1 / PD-L1	Agonizing PD-1 (PD-L1 fusion protein) suppresses pathogenic CD8^+^ T_RM_ activity and reverses tissue damage, whereas pharmacologically blocking PD-1 promotes cutaneous inflammation. PD-1 signaling suppresses BHLHE40, a transcription factor required for CD8^+^ T_RM_ persistence and mitochondrial fitness.	(72–74)
CD4^+^ t-cell help withdrawal from established CD8^+^ T_RM_	Chronic CD4 depletion does not reduce CD8^+^ T_RM_ numbers but lowers their CD103^+^ fraction, induces dependence on circulating-T-cell replenishment, and impairs recall IFN-γ. Evidence remains conflicting regarding CD4 dependence of established CD8^+^ T_RM_ during chronic disease.	(32, 75)

Strategies are grouped by the stage of CD8^+^ T_RM_ biology they primarily target (see **Figure 2**). "Key Biological Insight" summarizes what each strategy reveals about CD8^+^ T_RM_ behavior and specificity, not disease-specific clinical outcomes, which are detailed by disease in **Table 1**.

## Conclusion

Taken together, targeting CD8^+^ T_RM_ cells is not a straightforward therapeutic approach and requires careful consideration of several key factors. CD8^+^ T_RM_ cells are highly heterogeneous, in their phenotype, transcriptional programs, metabolic requirements, effector functions, and tissue-specific retention mechanisms, exhibiting diverse surface marker expression across diseases and anatomical sites (summarized in **Table 3**). Consequently, a therapeutic strategy effective in one disease context may not be applicable to another. In addition, many currently proposed targets lack strict specificity for pathogenic CD8^+^ T_RM_ cells, raising the possibility of unintended depletion of protective T_RM_ cell populations or other immune cell subsets in non-target tissues. Similarly, targeting transcriptional or metabolic programs may introduce broader off-target effects, as many of these pathways are pleiotropic and shared across multiple immune and non-immune cell types. Furthermore, interactions between different CD8^+^ T_RM_ cell subsets, as well as their crosstalk with other immune cell populations, add another layer of complexity. The long-lived nature of CD8^+^ T_RM_ cells also presents a challenge, as their accumulation during disease flare-ups may necessitate early intervention, along with sustained maintenance therapy. Considering these aspects, the identification of disease-promoting CD8^+^ T_RM_ subsets may not only facilitate the development of more targeted therapeutic strategies but also provide valuable prognostic biomarkers in chronic autoimmune and immune-mediated inflammatory diseases, and potentially predict treatment resistance.

**Table 3:** Comparative overview of tissue-specific CD8^+^ T_RM_ niches.

Tissue	Key retention signal(s)	Defining transcription factor(s)	Signature surface markers	Distinctive niche feature	Representative citation
Skin	TGF-β (integrin αvβ6/αvβ8-activated) → CD103/E-cadherin; IL-15 for survival and effector function	RUNX3; HOBIT/BLIMP-1; AhR	CD103, CD69, CD49a; CXCR3 (epidermis), CXCR6 (dermis)	Epidermal TGF-β-dependent CD103 imprinting; lipid uptake via FABP4/FABP5 sustains long-term survival	(1–3)
Lung	Constitutive epithelial CXCR6/CXCL16; Notch (γ-secretase-dependent)	BLIMP-1 (not HOBIT, unlike most other tissues); BHLHE40 (mitochondrial fitness)	CD69, CD103, ZNF683 (HOBIT, disease-associated); S1PR1-low	BLIMP-1, not HOBIT, is functionally required for TRM differentiation; γ-secretase inhibition disrupts maintenance of established T_RM_	(4–7)
Small Intestine	TGF-β with reduced IL-15 dependence relative to other tissues; CCR9/CCL25 homing	RUNX3; HOBIT/BLIMP-1; AhR (required for epithelial persistence)	CD103 and KLRG1 (mutually exclusive subsets), CD69, CD44, CXCR6	Reduced IL-15 dependence with strong epithelial TGF-β conditioning; CD103^+^ and KLRG1^+^ define functionally distinct T_RM_ subsets	(8–11)
Kidney	IL-7/IL-15-enriched interstitium; local antigen exposure alone sufficient for in situ differentiation	Not tissue-specifically defined; general KLF2 downregulation/S1PR1 loss applies	CD69, CD103 (acquired over time), granzyme B/K, PD-1, CD107a	Local antigen alone establishes T_RM_ despite loss of the corresponding circulating population	(12–17)
Liver (Parenchyma)	Species-divergent: LFA-1/sinusoidal patrolling (mouse) vs. CD103/E-cadherin (human disease); autocrine IL-2; hepatocyte-derived IL-7; IL-15/TGF-β induce differentiation	HOBIT (CIC–ETV5-regulated); BLIMP-1	CD69, CD103 (observed in human disease), CXCR6, CXCR3	Direct functional confirmation of CD103-dependent retention in humans is still lacking; steady-state mouse liver T_RM_ instead rely on LFA-1 for sinusoidal retention	(18–23)
Biliary Tract	NUDT1/PARP1 DNA-repair-dependent survival with TGFβ-Smad signaling	RUNX3; HOBIT	CD69, CD62L-negative, CD103, CD49a, CXCR6, CCR6, α4β7, CCR9, CXCR3	Unique DNA-repair/metabolic survival program (NUDT1-PARP1) — genetic or pharmacologic blockade eliminates the TRM population and alleviates disease	(24, 25)
Salivary Gland	High local TGF-β1–3; CXCR6/CXCL16 gradient recruits IL-15-primed circulating precursors	RUNX2/RUNX3; HOBIT/BLIMP-1	CD69, CD103 (two subsets of disputed relative pathogenicity), CXCR3, granzyme K/B	Granzyme K drives a non-apoptotic, cGAS-STING-mediated epithelial injury pathway, distinct from its complement-activating role in the CNS and its B-cell-promoting function in lupus nephritis	(26–33)
Pancreas	FABP4-dependent fatty acid oxidation sustains survival; T_RM_-derived CXCL10 recruits circulating CD8^+^ T cells	Not tissue-specifically defined	CD69, CD103 (acquired over time), FABP4	FABP4-dependent T_RM_ sustain diabetogenicity through a CXCL10 amplification loop; anti-CD69-mediated T_RM_ depletion delays diabetes onset.	(34, 35)
Joint / Synovium	TGF-β-responsive (type-17-like subset specifically); free-fatty-acid uptake; ST2 signaling	Cytotoxic subset: Eomes-high. Type-17-like subset: RORA, AHR, BATF, HOBIT. Peripheral-helper-like subset: TOX	CD69, CD103^+/-^, CXCR6, CD49a, PD-1, ST2, granzyme A/B/K, perforin	Same joint niche supports disease-specific transcriptional divergence — cytotoxic Eomes-high program in RA vs. type-17-like program in psoriatic arthritis — providing a potential mechanistic explanation for the differential efficacy of IL-17A blockade between the two diseases	(36–38)
Brain (CNS)	Treg-derived TGF-β (CD103 induction); glial PD-L1 → CD8^+^ PD-1 licensing; IL-33/ST2 (oligodendrocyte-derived)	TCF-1 (stem-like precursor); low T-bet/EOMES at baseline	CD103, CD69, PD-1, CTLA-4, CXCR6	CD4^+^ T cells are dispensable for initial T_RM_ establishment but required for differentiation into pathogenic effectors upon reactivation	(39–43)

Retention signals, transcription factors, and surface markers reflect the predominant patterns reported in the studies cited in this review for each tissue, in the context of the autoimmune or immune-mediated disease(s) discussed; within-tissue heterogeneity is addressed in the corresponding disease section(s) of the main text and in **Table 1**. "Distinctive Niche Feature" highlights the mechanism that most distinguishes each tissue's CD8^+^ T_RM_ biology from the others discussed here, and is intended to emphasize comparative biological principles rather than provide an exhaustive summary of each tissue.

### Limitations and future directions

Despite considerable progress in understanding CD8^+^ T_RM_ cell biology, several important questions remain unresolved. First, for most diseases, direct proof that CD8^+^ T_RM_ or T_RM_-like cells are pathogenically required, rather than simply present at the site of inflammation, is still lacking. Second, longitudinal sampling of the same lesion over time, rather than cross-sectional biopsies, is needed to determine whether CD8^+^ T_RM_ populations persist across disease flares and remission. Third, spatial transcriptomics and multiplex imaging approaches will help resolve how CD8^+^ T_RM_ cells are positioned relative to stromal niches, antigen-presenting cells, and target tissue structures, and how this spatial organization shapes their pathogenic potential. Fourth, single-cell TCR sequencing paired with transcriptomic and epigenetic profiling will help clarify clonal origins, the degree to which pathogenic CD8^+^ T_RM_ cell populations are oligoclonal and antigen-driven, and their relationship to circulating and central memory precursors. Addressing these questions will be critical for guiding research in autoimmune and immune-mediated inflammatory diseases.
